# Reconfigurable Inflatables Through Controlled Surface Crumpling

**DOI:** 10.1002/advs.202600074

**Published:** 2026-06-30

**Authors:** Yi Yang, Hye Jun Youn, Leon Kamp, Renate Sachse, Wenjie Li, Jose Vidal, Jin Feng, Martin Bechthold, Katia Bertoldi

**Affiliations:** ^1^ John A. Paulson School of Engineering and Applied Sciences Harvard University Harvard USA; ^2^ Graduate School of Design Harvard University Harvard USA; ^3^ Department of Civil and Environmental Engineering, TUM School of Engineering and Design Technical University of Munich Munich Germany

**Keywords:** crumpling, energy absorption, inflatables, multistability, reconfigurability

## Abstract

Inflatable structures offer remarkable versatility due to their compact storage and rapid deployment, making them ideal for lightweight, quickly assembled, and deployable applications. These structures are typically made from membranes that are nearly inextensible yet highly flexible. Upon inflation, the membranes avoid energy‐intensive stretching and instead deform primarily through bending, which results in the formation of localized surface crumples. While previous studies have largely focused on understanding the mechanics of crumple formation, here we take a different approach: we investigate how these surface crumples — traditionally viewed as a failure mode — can be harnessed to enable functionality. Specifically, we show how they can be used to design reconfigurable structures across scales and to develop advanced impact‐mitigation systems.

## Abstract 2

The study investigates how surface crumples, traditionally regarded as a failure mode, can instead be exploited to enable functionality. In particular, it shows how they can be leveraged to design reconfigurable structures across scales and to develop advanced impact‐mitigation systems.

## 1 Introduction

Due to their compact storage and ease of deployment, inflatable structures have enabled significant advances across a broad range of lightweight, deployable, and functional systems, including space habitats [1, 2, 3], airbags [4], soft robotics [5, 6, 7, 8, 9, 10, 11], and medical devices [12, 13]. Typically, the shape of an inflatable is predetermined by the arrangement of precursor sheets that are sealed together [14, 15, 16, 17], which limits the ability of these structures to adapt dynamically to changing tasks or environments once deployed.

Multistability has emerged as a powerful design strategy for enabling multifunctionality through in situ shape reconfiguration, significantly enhancing the adaptability and performance of engineered systems. This approach has been successfully applied across various domains, from metamaterials with reprogrammable mechanical properties [18] and energy‐trapping capabilities [19, 20], to soft robotic systems with preprogrammed directional motion [21] and autonomous control [22, 23]. In inflatable systems, multistability has been introduced by embedding origami‐inspired architectures into the membrane [24], as well as through square networks of inflatable slender pouches [25]. Furthermore, it has been shown that strategically patterning membranes with non‐inflating regions–realized through internal seals–can also give rise to multistable behavior [26, 27, 28].

Building on these advances, we present a mechanics‐based framework for embedding multistability into inflatable structures through the deliberate patterning of non‐inflating regions. Our study begins with a fundamental building block: a rectangular inflatable pouch with a pair of carefully positioned notches. These notches induce localized crumpling, enabling the transition from a monostable to a bistable mechanical response. By tessellating multiple such bistable units into periodic arrays, we design inflatable architectures capable of programmable and reconfigurable shape transformations at both centimeter and meter scales, offering new possibilities for deployable and adaptable architectural systems. Finally, we demonstrate that coupling multistability with the classical air‐cushioning effect significantly enhances the impact‐mitigation performance of inflatable structures. Collectively, these findings deepen our understanding of inflatable multistability and open new avenues for the design of adaptive, reconfigurable, and lightweight systems across a broad range of engineering applications.

## 2 Bistable Inflatable Building Block

**FIGURE 1:**
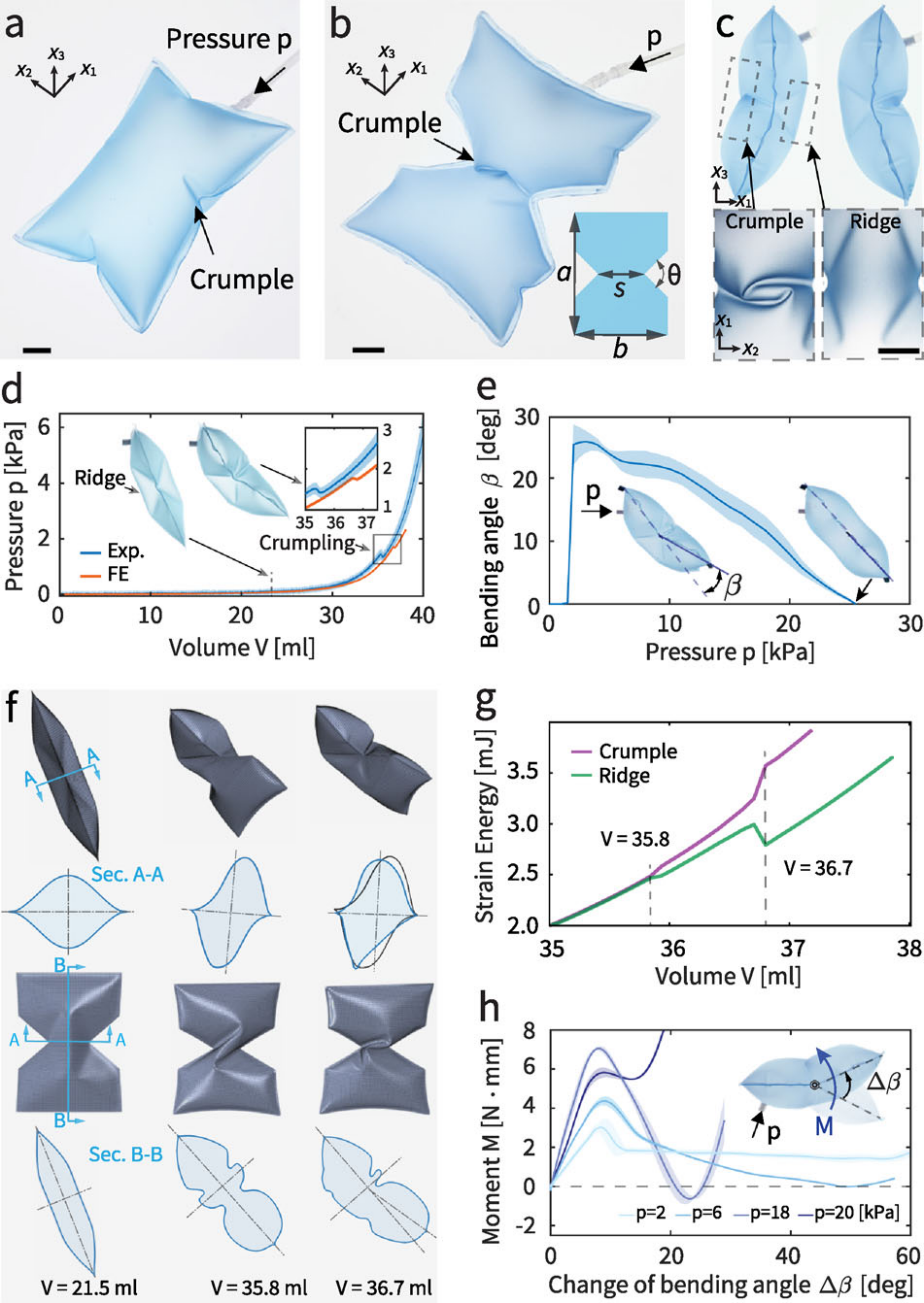
Bistable inflatable building block. (a) Crumples form near the sealing lines in a rectangular TPU inflatable. (b) In our building block, a rectangular inflatable with a pair of notches, crumples form across the region between the notches. (c) Side views of the building block at p=4 kPa, showing the formation of a bistable hinge, characterized by a crumple on one face and a ridge on the opposite face. The inflatable can be switched to a second stable configuration by reversing the side of the crumple and ridge. Zoom‐ins of the crumple and the ridge are also shown. (d) Pressure–volume curve of the building block. Insets show snapshots at different volumes and highlight the pressure drop at V=35.3 mL triggered by crumple formation. (e) Evolution of the bending angle as a function of pressure. (f) Numerical snapshots of the building block at V=21.5 mL, 35.8 mL, and 36.7 mL, showing two sequential symmetry‐breaking events. (g) Numerically predicted evolution of the strain energy in the two opposing faces of the inflatable building block as a function of volume. (h) Experimentally measured moment vs. change in bending angle for four different pressurization levels. Scale bars: 10 mm.

We investigate the behavior of macroscopic inflatables made with thermoplastic polyurethane (TPU) sheets with a Young's modulus E=30.2 MPa and Poisson's ratio ν=0.35, heat‐sealed along their boundaries using an ultrasonic welder mounted on a computer numerical control machine (see Section S1.1 for details). When an initially flat rectangular inflatable with length a=80 mm, width b=60 mm, and thickness t=0.15 mm is pressurized slowly, we observe the formation of crumples near the sealing lines (Figure 1a), consistent with previous observations for inflatables made from nearly inextensible but highly bendable films [29, 30, 31]. These crumples remain localized at the edges, and their position is largely dictated by unavoidable imperfections introduced during fabrication and loading.

To control the location of crumple formation, we introduce two mirrored notches with an opening angle $\theta =90^{\circ }$ at the center of the inflatable, reducing its central width to s=30 mm. In this case, a crumple, spanning the region between the two notches, consistently forms on one of the faces of the inflatable at a pressure p=1.7 kPa (Figure 1b). This crumple breaks the symmetry between the two faces and initiates the formation of a localized hinge that causes bending of the inflatable (Figure 1c and Video S1).

The crumple and the resulting hinge emerge through a complex sequence of deformations during inflation. Initially, two ridges appear to be observable at the notch locations for a volume of the air cavity V≈15 mL (corresponding to a pressure p≈0.06 kPa). As inflation progresses, the inflatable begins to twist about its longitudinal axis at V=24.8 mL. At V=35.3 mL, one of the ridges transforms into a crumple that spans the central width s (Figure 1d). This transition creates a marked asymmetry between the two faces of the inflatable, where one side retains a ridge, while the other forms a crumple, resulting in a localized hinge that drives bending (Figure 1c). Notably, the formation of the crumple is accompanied by a sharp pressure drop, suggesting that it is associated with a snapping instability (Figure 1d). Furthermore, we find that the crumpling‐induced hinge angle β reaches its maximum value of $25.6^{\circ }$ near the onset of the crumpling and gradually decreases as the pressure increases, eventually vanishing as the inflatable flattens out completely at p=25 kPa (Figure 1e).

To gain deeper insight into the experimental observations, we simulate the nonlinear deformation behavior of the inflatable under pressurization using Finite Element (FE) analysis with the commercial software ABAQUS/Standard. The geometry is discretized using 4‐node linear shell elements, and the mechanical response of the TPU sheets is modeled with a first‐order Ogden hyperelastic material model. Inflation is simulated via an implicit dynamic analysis, in which the volume of the internal cavity V is gradually increased (see Section S2.5 for details). The numerically predicted evolution of pressure p as a function of volume V closely matches the experimental measurements (Figure 1d), confirming the accuracy of our simulations. Notably, the FE simulations capture the pressure drop associated with the onset of crumple formation at V=35.2mL, and successfully reproduce the complex deformation observed in the experiments (Figure 1f). Initially, two ridges begin to form in the notched region, and the inflatable deforms symmetrically up to V=21.5 mL. At this point, a symmetry‐breaking event occurs, initiating a twisting deformation until reaching a volume of V=35.8 mL (see cross‐section A– A in Figure 1f). As the volume increases further to V=36.7 mL, a second symmetry‐breaking transition occurs: the ridge on one face collapses into a crumple (cross‐section B– B in Figure 1f), leading to the emergence of a localized hinge that produces a bending angle β. Notably, the crumple formation also redistributes the strain energy between the two faces of the inflatable (Figure 1g). As previously reported for stretched elastic sheets [29, 32], the strain energy becomes localized on the crumpled face, while it decreases on the opposite face, which retains the ridge.

The inflatable can be switched to a second stable configuration by applying an external moment, reversing the side of the crumple and ridge. This causes the inflatable to bend in the opposite direction (Figure 1c and Video S1). To quantify this bistable behavior, we experimentally measure the moment M required to induce a change in the bending angle Δβ at four different pressures (Figure 1h and Section S2.1.3 for details). Across all pressures, we observe that M increases with increasing Δβ before the collapse of the ridge into a crumple. Then, M decreases as the ridge collapses into a crumple on one side, while the crumple on the opposite side transitions into a ridge. Notably, at pressures of p=6 and p=18 kPa, there is a brief interval where the moment becomes negative, indicating bistability of the hinge. For the inflatable studied here, bistability occurs within a pressure range of 2 to 18 kPa. This bistable behavior is robust and persists across a wide range of geometric parameters, including a, b, s, t, and θ. However, it is worth emphasizing that changes in geometric parameters affect the bending angle achieved by the crumple‐induced hinges (see Section S2.2 for details).

## 3 Multistable Inflatable Tessellations

**FIGURE 2:**
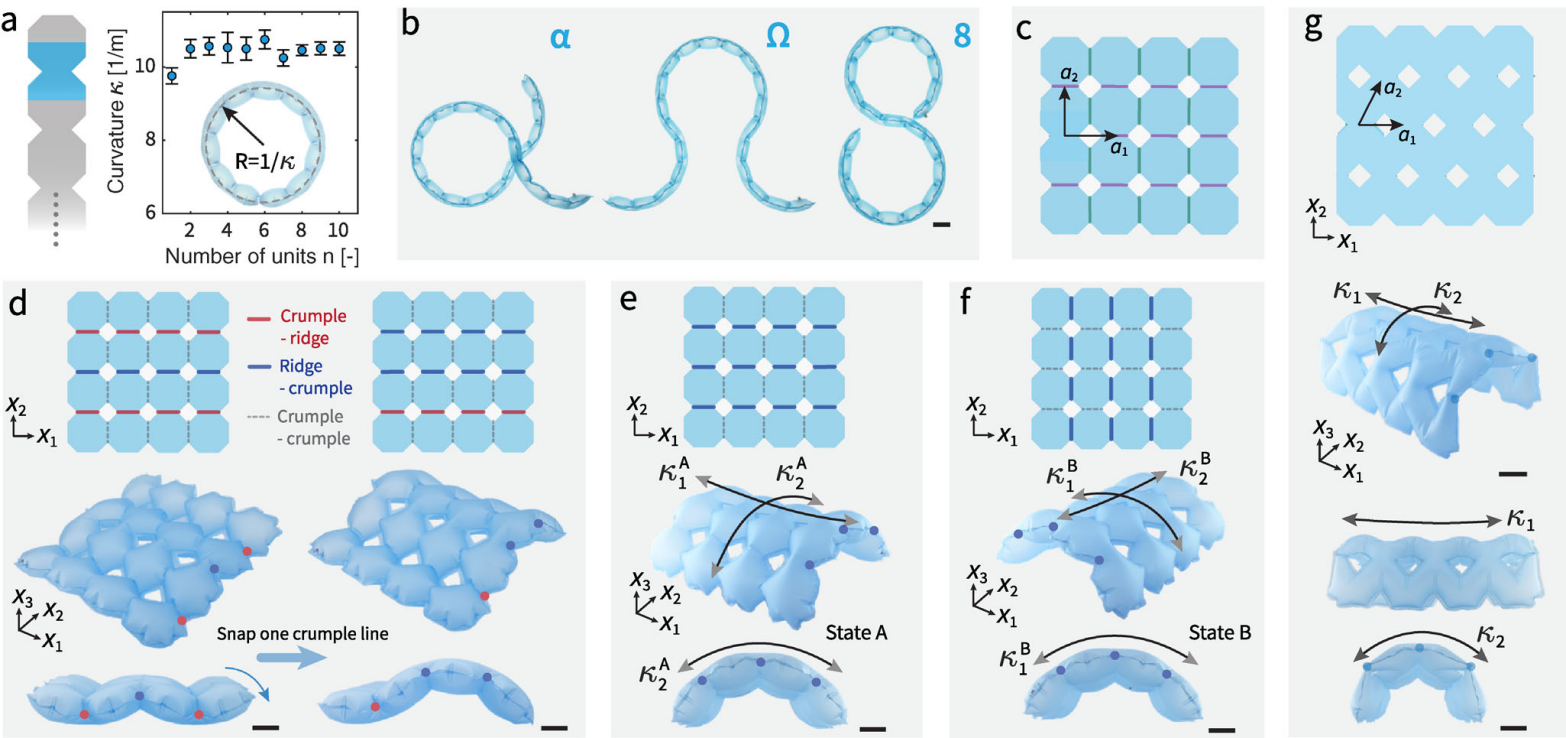
Multistable inflatable tessellations. (a) Measured curvature of one‐dimensional arrays of building blocks inflated at p=6 kPa as a function of the number of units. The error bars represent the standard deviation obtained from repeated measurements on three fabricated samples with identical geometry. (b) Images showing three distinct stable configurations supported by a 1D array composed of 20 building blocks. (c) Schematic of a two‐dimensional square tessellation constructed from the inflatable building block. Green and purple lines indicate the two perpendicular families of crumple lines. (d) Images of two stable configurations supported by a 4×3 square tessellation. Red, blue, and grey lines in the schematics above indicate the distribution of crumple‐ridge, ridge‐crumple, and crumple–crumple pairs that give rise to these configurations. (e) Image of the stable configuration supported by a 4×3 square tessellation where all ridge‐crumple lines are oriented along the $a_{1}$ direction. (f) Image of the stable configuration supported by a square 4×3 tessellation where all ridge‐crumple lines are oriented along the $a_{2}$ direction. (g) Image of a stable configuration supported by a triangular tessellation of the considered inflatable building block. Scale bars: 2 cm.

Bistable inflatable building blocks, such as the one shown in Figure 1, can be used to construct multistable inflatables that can switch between various complex shapes. In Figure 2, we present results for tessellations of a building block with a=b=60 mm, s=30 mm and $\theta =90^{\circ }$. Note that, to enable square tessellations, we select b=a which is different from that considered in Figure 1, but this change does not have a significant impact on the mechanical behavior of the building block for shape‐shifting. As a starting point, we examine a one‐dimensional array of these building blocks. Upon inflation, pairs of crumples and ridges that induce bending emerge at each notch, giving the structure a curved shape (Figure 2a). Importantly, the curvature of the inflatable, κ, is determined by the bending angle β of every crumple‐induced hinge and only minimally influenced by the number of units n in the chain (Figure 2a). Each hinge formed in the chain retains its bistable characteristics over nearly the same pressure range as the inflatable building block. Thus, with n building blocks in series, the inflatable can switch between $2^{n-2}+2^{n/2-1}$ distinct stable configurations when n is an even number, and $2^{n-2}+2^{(n-3)/2}$ distinct stable configurations when n is an odd number. In Figure 2b, we show three of these stable configurations for a chain of n=20 units at p=6 kPa (see also Video S2).

Next, we investigate the behavior of two‐dimensional tessellations constructed from our inflatable building block. When arranged in a rectangular tiling defined by the lattice vectors $a_{1}=\left[b,\;0\right]$ and $a_{2}=\left[0,\;a\right]$, the notches align to form a regular grid of diamond‐shaped non‐inflating regions. In a 4×3 tessellation of the considered building block with a=b and $\theta =90^{\circ }$, a 3×3 array of square non‐inflating regions emerges (Figure 2c). Upon inflation, each non‐inflating region gives rise to four crumples – one at each of its corners – that extend toward the corners of adjacent non‐inflating region. As a result, the non‐inflating regions collectively give rise to three crumple lines aligned with $a_{1}$ and three more aligned with $a_{2}$ (highlighted by green and purple lines in Figure 2c). Crucially, the continuous membrane introduces mechanical coupling between adjacent crumple lines, constraining the possible deformation modes and resulting in two distinct configurations: (i) *Ridge‐crumple pairs*, in which a crumple on one face aligns with a ridge on the opposite face to form a bistable hinge– similar to what is observed in individual building blocks; (ii) *Crumple‐crumple pairs*, in which crumples appear on both faces, locally softening the inflatable structure without producing rotation or bistable behavior (gray dashed lines in Figure 2d). Notably, the structure shown in Figure 2c supports ridge‐crumple pairs only along one family of parallel lines at a time. This limitation is reminiscent of rigid origami, where two orthogonal creases that intersect at a vertex cannot be folded simultaneously [33].

In Figure 2d, we show two stable configurations supported by the inflatable considered (see also Video S2). In both configurations, ridge–crumple pairs align with the lattice vector $a_{1}$, while crumple–crumple pairs align with $a_{2}$. This results in three lines of bistable hinges aligned with $a_{1}$. When the crumples are located on the top face, they form valley folds (highlighted by red lines in Figure 2d); when they are on the bottom face, they form mountain folds (highlighted by blue lines in Figure 2d). Notably, by applying a mechanical load, we can switch the positions of the crumples and the ridges, effectively transforming a valley fold into a mountain fold and vice versa. These transformations are also captured in FE simulations, see Section S3.6 for further details.

When all three bistable hinges bend in the same direction, the inflatable adopts an arch‐like shape (Figure 2e). Due to the symmetry of the square tiling, bending either one set of parallel hinges or its perpendicular counterpart results in structurally identical shapes, labeled as State A and State B in Figure 2e,f, respectively. However, the increased compliance of the two‐dimensional inflatable reduces the overall curvature of the arch compared to that of the corresponding one‐dimensional tessellation. While the 1D configuration exhibits a curvature of κ=10.58 1/m, the 2D inflatable structure shows a reduced primary curvature of $\kappa_{2}^{A}=7.04$ 1/m and $\kappa_{1}^{B}=6.49$ 1/m in State A and State B, respectively. Additionally, this coupling induces a small negative curvature in the orthogonal direction, measured as $\kappa_{1}^{A}=-1.09$ 1/m or $\kappa_{2}^{B}=-1.01$ 1/m for State A and State B, respectively.

It is important to note that the spatial arrangement of the building blocks plays a critical role in determining the locations of crumple‐induced bistable hinges and, ultimately, the range of accessible stable configurations. In Figure 2g, we focus on a triangular tiling of the building block, defined by the lattice vectors $a_{1}=\left[b,\;0\right]$ and $a_{2}=\left[0.5b,\;0.866b\right]$, which generates a triangular array of square non‐inflating regions. Upon inflation, crumples form at all corners of the non‐inflating regions. However, only those aligned with $a_{1}$ can form continuous crumple lines. As a result, this inflatable supports only parallel lines of bistable hinges along the $a_{1}$ direction (Video S2). In Figure 2g, we present a stable configuration in which all three hinge lines bend in the same direction. In this state, the inflated structure assumes a shape reminiscent of a cylindrical shell, characterized by a primary curvature of $\kappa_{2}=9.22$ 1/m and a much smaller curvature in the orthogonal direction, $\kappa_{1}=-0.62$ 1/m.

**FIGURE 3:**
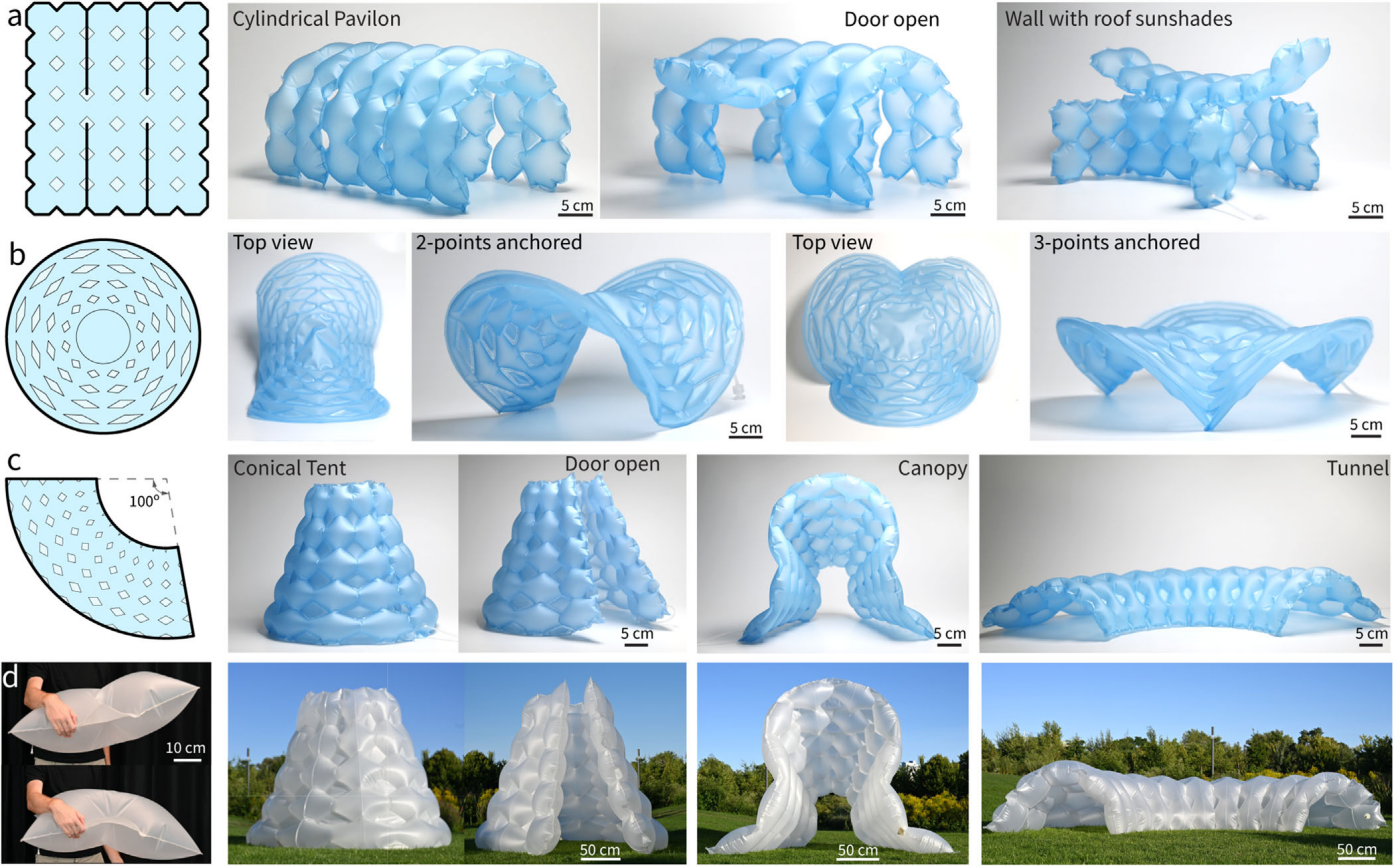
Reconfigurable multistable inflatables across scales. (a) Images of three stable configurations supported by a 6×7 square array of building blocks with four cuts aligned along the $a_{2}$ direction (indicated by thick black lines). (b) Top and side views of two stable configurations supported by a disk tessellated with the building block. (c) Images of three stable configurations supported by a 10×5 array of diamond‐shaped non‐inflating regions arranged on an annular sector. (d) Snapshots of the bistable meter‐scale building block, along with a stable configuration of a meter‐scale inflatable identical to that in panel c, but the in‐plane geometry is scaled by a factor of 10.

The range of stable shapes achievable with inflatable tessellations extends far beyond those shown in Figure 2. As an example, in Figure 3a, we expand the tessellation from Figure 2c into a 6×7 array and introduce four cuts aligned with $a_{2}$ (indicated by black lines in Figure 3a). These cuts reduce hinge coupling in the direction perpendicular to them, effectively dividing a single hinge line along $a_{1}$ into three independent hinges (Figure S19). This increases the number of accessible configurations and enables the creation of tunnel‐like structures with openings that can be switched between closed and open states (Figure 3a and Figure S19 and Video S3).

Conversely, hinge coupling is enhanced when the building block is tessellated on a disk forming a closed loop. In Figure 3b, we consider a circular array of building blocks, which results in 48 diamond‐shaped non‐inflating regions, dimensioned to maintain a uniform corner‐to‐corner spacing of s=15 mm in both the radial and tangential directions (Figure S20). In this configuration, geometric frustration arises because the hinges along the tangential direction are not aligned but are instead angled to follow the circular geometry (see Section S3.4 for details). Consequently, bistable line hinges in this inflatable structure can only form in the radial direction, resulting in an undulating saddle shape. The number of undulations can be controlled by adjusting the boundary conditions during inflation. At the points along the boundary where the sheet is held, the hinge line leads to valley folds, whereas the other hinge lines form mountain folds resulting in a perimeter that follows a continuous smooth curve. This makes it possible to increase the number of undulations from 2 to 3 by increasing anchored points (Figure 3b and Figure S22 and Video S3). Therefore, control of boundary conditions during inflation provides a means to program desired shape without manual handling (see Section S3.3 for details). Additionally, as with all other structures, the number of undulations can be varied by manually applying mechanical loads to the structure after inflation to switch radial hinge (see Video S3).

In Figure 3c, we show a 10×5 array of diamond‐shaped non‐inflating regions arranged on an annular sector with a central angle of 100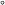 and designed to maintain a constant corner‐to‐corner spacing of s=30 mm (Figure S21). Similarly to the square array, in this configuration, bistable hinge lines can form in both the radial and tangential directions, though not simultaneously. This behavior indicates that the compliance of the inflatable can partially overcome geometric frustration, enabling the formation of hinges along the tangential direction when geometric compatibility is low. When hinge lines form along the radial direction, the inflatable adopts a truncated conical shape, and the inflatable is designed so that if all radial crumples fold in the same direction, it laterally closes. However by selectively flipping the bending direction of the radial hinge lines, it is possible to create an opening into a conical shape or to form various undulating profiles, including the canopy shape shown in Figure 3c. Notably, the compliance of the inflatable enables bistable hinge lines to reorient from the radial to the tangential direction, forming curved hinge lines reminiscent of curved‐crease origami [34, 35]. In this configuration, the inflatable adopts a tunnel‐like shape (Figure 3c and Video S3).

The ability to create lightweight inflatable structures that can be reconfigured into tunnels and canopies with adjustable openings presents exciting opportunities for designing large‐scale, reconfigurable systems. To demonstrate this potential, we scale up the design shown in Figure 3c and fabricate it at the meter scale. In Figure 3d, we present snapshots of the bistable building block scaled by a factor of 10 with dimensions a=80 cm, b=60 cm, s=30 cm and $\theta =90^{\circ }$, and fabricated from TPU with a thickness of t=0.7 mm. Upon inflation, a bistable hinge consistently forms between the two notches. However, achieving airtight seals in thicker TPU films can weaken the bond strength, limiting the maximum internal pressure to p=4 kPa. Furthermore, scaling up introduces new fabrication challenges as limits in material size and fabrication bed require fabricating the inflatable in sectors with the same strategy as centimeter‐scale samples and then welding them together with an industrial heat sealer (see Section S1.2 for details). As shown in Figure 3d, the meter‐scale inflatable can be reconfigured into multiple stable configurations, transitioning from a tent to a canopy to a tunnel (see also Video S4). While large‐scale inflatables –particularly in architectural applications – are typically designed to maintain wrinkle‐ or crumple‐free surfaces, this design intentionally leverages crumples to generate localized stability, enabling the formation of a reconfigurable and stable structure at meter‐scale.

Beyond enabling shape‐shifting capabilities, multistable inflatables present promising opportunities for developing lightweight structures with enhanced mechanical performance. Drawing inspiration from recent studies that highlight the use of snapping instabilities to improve energy absorption in lattice structures [19, 20, 36], we demonstrate that our multistable inflatables can substantially increase the energy dissipation capacity of inflatable‐based shock absorbers. These shock absorbers are especially appealing due to their low weight, deployability, and reusability [37], making them suitable for a broad range of impact mitigation applications, such as vehicle airbags [38] and airbag landing mats [37, 39]. In these systems, energy is absorbed through the compression and redistribution of air within internal chambers upon impact, with the compressed air acting simultaneously as a spring and a damper to cushion the blow and reduce the force transmitted to the protected object [40]. Importantly, in our multistable inflatables, energy absorption arises not only from air redistribution but also from the snapping of bistable hinges, which acts as an additional dissipation mechanism.

**FIGURE 4:**
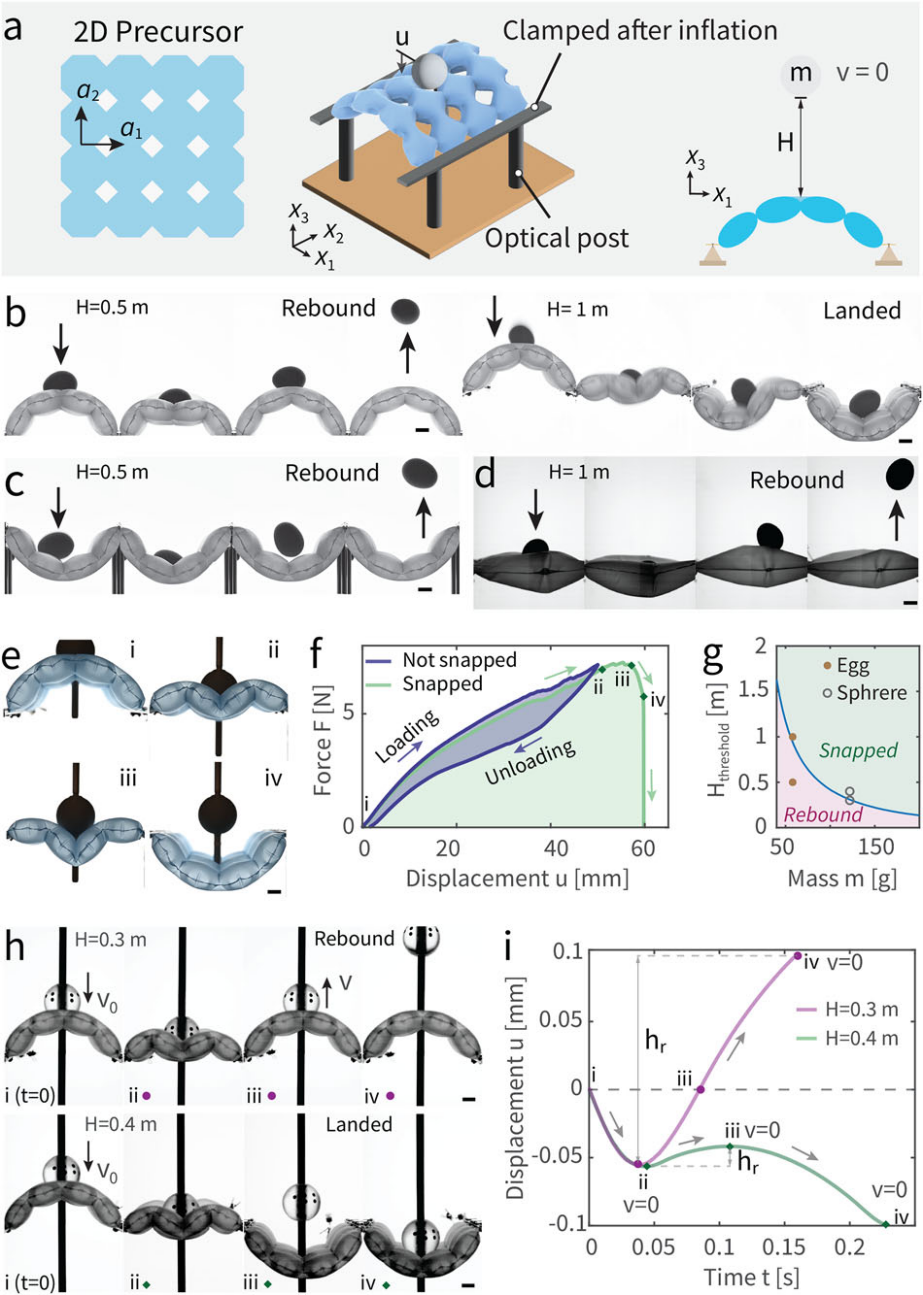
Multistable inflatables for impact mitigation. (a) Schematic of the experimental setup. (b) Snapshots of an egg dropped onto the multistable inflatable (initially in the upward‐curved configuration) from heights of H=0.5 m (left) and H=1.0 m (right) above its apex. (c) Snapshots of an egg dropped onto the multistable inflatable (initially in the downward‐curved configuration) from a height of H=0.5 m. (d) Snapshots of an egg dropped onto a square inflatable pouch– having the same dimensions and air volume as the multistable structure– from a height of H=1.0 m. (e) Snapshots and (f) force–displacement curve from quasi‐static indentation tests, in which the multistable inflatable is loaded at its center using a rigid sphere. (g) Numerically predicted minimum drop height required to trigger snapping as a function of the impacting object's mass. (h) Snapshots of a rigid sphere dropped from H=0.3 m and H=0.4 m. (i) Recorded trajectory of a rigid sphere dropped from H=0.3 m and H=0.4 m. Scale bars: 2 cm.

To demonstrate the enhanced energy dissipation provided by multistability, we carried out a series of drop tests using raw eggs weighing between 46 and 58 g. In all tests, the inflatable structures were suspended in the air with two opposite edges clamped (Figure 4a). Figure 4b shows snapshots from a test where the egg was dropped onto a multistable inflatable, which is identical to the one shown in Figure 2d, pressurized to 6 kPa and configured in its upward‐curved, stable arch‐like state. When the egg was released from a height of 0.5 m above the apex of the inflatable (Figure 4b left panel and Video S5), the impact energy was insufficient to trigger a transition to the structure's inverted stable state. Although the air cushion mitigated the initial impact, the inflatable quickly returned to its original shape, causing the egg to rebound and fall to the ground, where it broke. In contrast, when the egg was dropped from a height of 1 m, the impact energy was sufficient to trigger the snapping transition to the inverted configuration (Figure 4b right panel and Video S5). In this case, the structure remained in its new stable state after impact, providing a soft landing platform that prevented the egg from bouncing and allowed it to remain intact. It is important to emphasize that the survival of the egg is not simply due to the inflatable adopting a downward‐curved shape. To illustrate this, we performed a control test in which an egg was dropped from a height of 0.5 m onto the inflatable initially configured in its downward‐curved, stable cylindrical state. In this case, no snapping transition was triggered upon impact, and the egg rebounded off the surface and fell to the ground, where it broke (Figure 4c and Video S5), highlighting that the snapping mechanism plays a critical role in enabling the protective effect. Finally, in Figure 4d, we show snapshots from an additional control experiment, where the egg was dropped onto a square inflatable pouch with the same dimensions and air volume as the multistable inflatable. Although the pouch provided an air cushion, it responded similarly to the multistable inflatable in the absence of snapping: it quickly returned to its original shape after impact, causing the egg to rebound and break. These comparisons highlight the significance of the snapping transition in enhancing the impact energy dissipation of inflatables, even when constrained by limited fabrication materials and air volume.

To quantitatively assess the contribution of snapping to energy absorption, we performed quasi‐static displacement‐controlled indentation tests on the considered multistable inflatable pressurized to 6 kPa. In these tests, a 3D printed rigid solid sphere with a diameter of 60 mm was pressed against its center while the two lateral edges were clamped (Figure 4a and Video S5). Applying a displacement of u=59.8 mm causes the central hinge line to snap from a mountain fold to a valley fold, followed in quick succession by the snapping of the other two hinge lines, ultimately inverting the arch structure (Figure 4e,f). Notably, because the sphere is not attached to the inflatable, lifting it leaves the structure in its inverted configuration. If the loading is stopped before snapping occurs, by applying a displacement of u=50 mm, the inflatable returns to its original configuration upon unloading, and the energy used to mitigate the impact through dissipation in the cycle is 58.7 mJ (shaded purple area in Figure 4f). In contrast, when a larger displacement u=59.8 mm is applied, which is sufficient to trigger the snapping of all three hinge lines, the energy for impact mitigation increases markedly to 271.2 mJ (shaded green area in Figure 4f).

The minimum drop height required to trigger snapping along all hinge lines for a given mass can be estimated using a reduced‐order model. In this model, the multistable inflatable is represented by a nonlinear spring in parallel with a linear dashpot. By integrating the equations of motion for this single‐degree‐of‐freedom mass–spring–damper system, we can estimate the drop height required to generate a force in the nonlinear spring sufficient to trigger snapping (see Section S3.5 for details). For instance, for a 3D‐printed rigid sphere with a mass of 122 g and a diameter of 60 mm, the model predicts a threshold drop height of $H_{\text{threshold}}=0.31$ m to induce full snapping (Figure 4g). To validate this prediction, we conducted two drop tests from heights of H=0.3 m and H=0.4 m. As predicted, the 0.3 m drop did not provide enough impact energy to trigger the transition to the inverted stable state, and the sphere rebounded (Figure 4h). In contrast, the 0.4 m drop successfully triggered snapping, causing the structure to transition to its inverted stable state and significantly reducing rebound. To better quantify the dynamics of the two experiments, with and without snap‐through, we monitor the displacement u of the sphere and define the rebound height of the sphere, $h_{r}$, as the vertical displacement between the instant at which the sphere first comes to rest (i.e., when its velocity vanishes) and its subsequent rebound peak. As shown in Figure 4i, when snap‐through is not triggered (purple curve), the rebound height is $h_{r}=0.152$ m. In contrast, when snap‐through occurs (green curve), the rebound height is dramatically reduced to $h_{r}=0.015$ m. This pronounced reduction in rebound height demonstrates that the snap‐through of the pouch dissipates a significant portion of the sphere's kinetic energy, thereby fundamentally altering the collision outcome compared to cases without snap‐through.

## 4 Conclusion

In summary, we have shown that crumple formation in inflatable structures can be strategically leveraged to achieve multistability. In particular, through a combination of experiments and simulations, we demonstrated that introducing notches into a rectangular inflatable enables controlled crumple formation and the emergence of bistable hinges. Building on this insight, we designed multistable inflatable tessellations that give rise to reconfigurable structures across length scales. Finally, we showed that these multistable inflatables offer enhanced energy dissipation capabilities, highlighting their potential for impact‐mitigation applications.

In our system, multistability emerges from the controlled formation of surface crumples that develop intrinsically within the inflatable structures upon inflation, without the need for embedded hinges, fold lines, or auxiliary structural components. This material‐level encoding of multistability offers distinct advantages for large‐scale deployable systems, such as simplified fabrication, reduced assembly complexity, and post‐fabrication programmability, albeit at the expense of load‐bearing capacity. Given the widespread availability of fabrication technologies, our approach is readily scalable to meter‐scale systems suitable for diverse applications, including reconfigurable pavilions and impact‐mitigation devices. Additionally, the proposed multistable inflatables open exciting opportunities for advancing human–computer interfaces, including applications in haptic feedback [41, 42, 43] and smart wearables [44, 45]. As a proof of concept, we demonstrate that our inflatable building block can act as a switch for a desktop lamp–without any electronic components embedded within the inflatable itself (see Section S2.4 and Video S1). The performance of these multistable structures could be further enhanced by integrating responsive materials for programmable actuation, paving the way for a new generation of lightweight, reconfigurable, and multifunctional smart wearable devices, adaptive architectural systems [46, 47], and inflatable structures for deployable habitats and space debris mitigation.

## 5 Materials and Methods

Details of the fabrication methods are provided in Section S1. The experimental and numerical procedures used to characterize the mechanical response of the inflatable building blocks are described in Section S2 and Section S3 presents the experimental characterization of one‐ and two‐dimensional multistable inflatable tessellations.

## Author Contributions

Y.Y. H.Y. and K.B. proposed and developed the research. H.Y. and Y.Y. designed and fabricated the centimeter‐scale inflatables. H.Y. and L.K designed and fabricated the meter‐scale inflatable structures. Y.Y. and L.K. designed experiments and performed data analysis. Y.Y. W.L. J.V. and L.K. conducted the experiments. R.S. and J.F. performed the numerical simulations. Y.Y. H.Y. L.K. R.S. and K.B. wrote the paper. M.B. and K.B. supervised the research.

## Conflicts of Interest

The authors declare no conflicts of interest.

## Supporting information


**Supporting File 1**: advs76279‐sup‐0001‐SuppMat.pdf.

**Supporting File 2**: advs76279‐sup‐0002‐VideoS1.mp4.

**Supporting File 3**: advs76279‐sup‐0003‐VideoS2.mp4.

**Supporting File 4**: advs76279‐sup‐0004‐VideoS3.mp4.

**Supporting File 5**: advs76279‐sup‐0005‐VideoS4.mp4.

**Supporting File 6**: advs76279‐sup‐0006‐VideoS5.mp4.

## Data Availability

The data that supports the finding of this study are available in Supplementary Information.
