# Supplementary material for: Reconfigurable Inflatables Through Controlled Surface Crumpling
Source: Adv Sci (Weinh). 2026 Jun 30;13(47):e00074. doi: 10.1002/advs.202600074 (PMC13336512; doi:10.1002/advs.202600074)
Supplement: Supplementary file 1 [file ADVS-13-e00074-s006.pdf]

Supplementary Materials

Reconfigurable Inflatables through Controlled Surface Crumpling

Yi Yang, Hye Jun Youn, Leon Kamp, Renate Sachse, Wenjie Li, Jose Vidal, Jin Feng, Martin Bechthold, Katia Bertoldi

E-mail: bertoldi@seas.harvard.edu

This PDF file includes:

Supplementary text
Figs. S1 to S28
Captions for Movies S1 to S5
References for SI reference citations

Other supplementary materials for this manuscript include the following:

Movies S1 to S5

Supporting Information Text

S1. Fabrication

This section describes the fabrication of the inflatables at both the centimeter-scale and meter-scale.

S1.1. Fabrication of centimeter-scale inflatables. All centimeter-scale inflatables are fabricated using thermoplastic polyurethane (TPU) films (Vendor: *Jinryangulraetan*, South Korea). The fabrication process employs an ultrasonic welder (BAOSHISHAN Ultrasonic Plastic Welder 500W) mounted on a computer numerical control (CNC) cutting machine (Zünd G3 M-1600 cutter, Zünd Systemtechnik AG), equipped with a Z-axis slider (FESTO Minislides DGSL). Detailed fabrication steps are outlined below and illustrated in Fig. S1:

- **Step 1:** A line drawing of the desired 2D welding pattern is prepared using CAD software. A 10 mm opening is intentionally left along one edge of the pattern to facilitate valve insertion in later steps.
- **Step 2:** Two sheets of TPU film are stacked on the CNC machine bed, separated from the ultrasonic welder by a protective rubber layer to prevent heat damage.
- **Step 3:** A Teflon sheet is placed over the TPU layers, and the welding pattern is traced using the ultrasonic welder.
- **Step 4:** The welded pouch is manually cut from the sheet using scissors.
- **Step 5:** A valve is inserted through the pre-formed opening. The valve is pushed through a small puncture made with a needle; the puncture is deliberately smaller than the valve diameter to ensure a tight seal. Each valve is 3D-printed with dimensions: height 5 mm, hole diameter 1 mm, and cylinder width 2 mm.
- **Step 6:** The gap around the valve is sealed using a linear impulse heat sealer (model FS-400), operated at a setting between level 2 and 5 depending on the TPU thickness.
- **Step 7:** The valve is connected to a pressure regulator (*Fluigent LineUp™ Push-Pull*) to inflate the pouch.

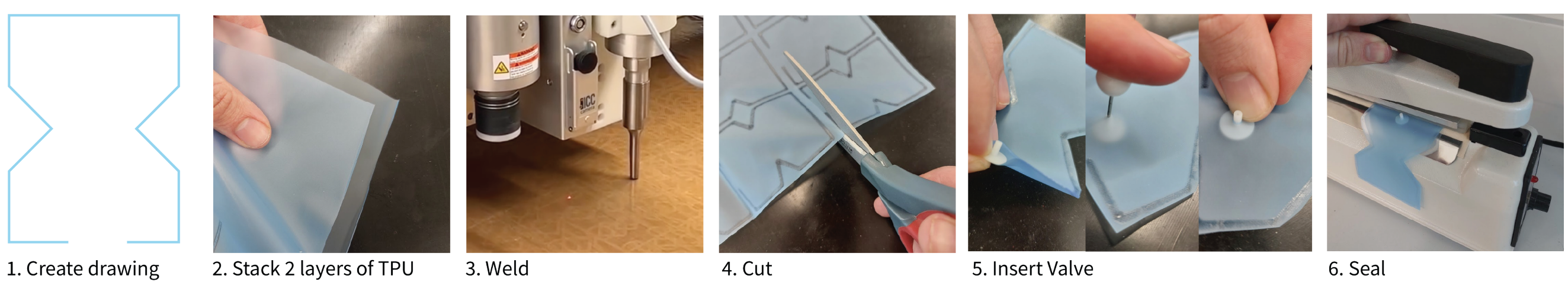

Fig. S1. Fabrication of centimeter-scale inflatables. Snapshots of the steps required to fabricate our centimeter-scale inflatables.

S1.2. Design and fabrication of the meter-scale inflatable. To demonstrate that the design principles identified in this work can be extended to human-scale structures, we fabricated the large-scale reconfigurable structure shown in Fig. 3d of the main text. The overall dimensions of this structure were constrained by the roll width of the TPU material (1100 mm). Accordingly, each segment was scaled to a width of 1050 mm, allowing a 50 mm tolerance to accommodate fabrication errors. At this scale, each segment has a length of 3400 mm and inflates into a conical shape with a height of approximately 3000 mm, which is sufficient to fully encapsulate a person. Based on these planar dimensions, the in-plane dimensions of the inflatable building block shown in Fig. 1 of the main text were uniformly scaled by a factor of approximately 10. With respect to material thickness, increasing the TPU thickness t leads to a heavier but more stable structure. As a trade-off between weight and stability, we selected a thickness of $t = 0.7$ mm. A building block with $t = 0.7$ mm and in-plane dimensions ten times larger than those considered in Fig. 1 is expected to be bistable, as this corresponds to a uniform scaling of the unit cell with thickness $t = 0.07$ mm reported in Fig. S10C (blue curve). At this thickness, the total mass of the structure remains manageable (approximately 42 kg), allowing it to be carried by two people.

The large-scale inflatable presented in this study is fabricated from 63 meters of 1.1-meter-wide TPU film with a thickness of 0.7 mm, selected to increase the energy barrier for switching between stable configurations. This added thickness enables the inflatable to remain bistable under the increased weight of the large unit cell. While the fabrication process follows the same basic principles as the centimeter-scale samples, several additional steps are required to accommodate the size constraints of the CNC bed (1330 mm wide × 1600 mm long) and the width of the TPU roll (1100 mm). To address these limitations, the inflatable is fabricated in 11 separate segments (Fig. S2).

Each segment contains a central column of four quadrilateral welds and two triangular welds, carefully aligned so that seams do not interfere with the welded patterns. Additionally, each weld pattern is repeated with a 6 mm offset to increase

the mechanical strength of the inflatable. Segments are scaled to 1050 mm in width to fit within the TPU sheet, allowing for fabrication tolerances, and each segment is 3400 mm in length. Since the CNC bed's working area is limited to 1660 mm, each segment is produced in six sequential stages following the process below (Fig. S3):

- **Step 1:** Two layers of TPU film are stacked on the CNC bed, and a layer of rubber is placed underneath to protect the film from the heat of the ultrasonic welder. A test run without welding is performed to verify the proper alignment and positioning of the material.
- **Step 2:** A Teflon sheet is placed over the TPU and the designated pattern is welded (power setting: level 8–9). Additionally, four 5 mm diameter registration dots are welded near the top edge of the CNC bed to serve as alignment markers.
- **Step 3:** The Teflon sheet is removed, and black adhesive markers are placed on top of the welded registration dots to improve visibility for alignment.
- **Step 4:** The partially welded TPU film is slid downward until the registration dots are aligned with the bottom edge of the CNC bed. The material is taped in place, and the CAD pattern is aligned to the new position using the CNC machine's registration feature (*Zünd*).
- **Step 5:** Steps 2–4 are repeated to weld the second and third sections of the segment, with the film shifted and aligned using the registration dots at each stage.
- **Step 6:** The ultrasonic welder is replaced with a cutting blade on the CNC machine, and the completed segment is cut from the TPU sheet using the same alignment method.
- **Step 7:** The seams of two finished segments are aligned and welded together along both sides using an industrial heat sealer.
- **Step 8:** The outer edges of the assembled inflatable are sealed, and a boat valve is inserted.
- **Step 9:** The completed inflatable is inflated to a pressure of 4 kPa using an air pump (*Scoprega GE OV 10/120*). Note that the large-scale structure was inflated to 4 kPa rather than 6 kPa, as the longer seams at this thickness proved less reliable during fabrication. Operating at this reduced pressure introduces a safety margin that slightly reduces structural stability, while ensuring the formation of crumples at pressures that do not compromise the welded seams.

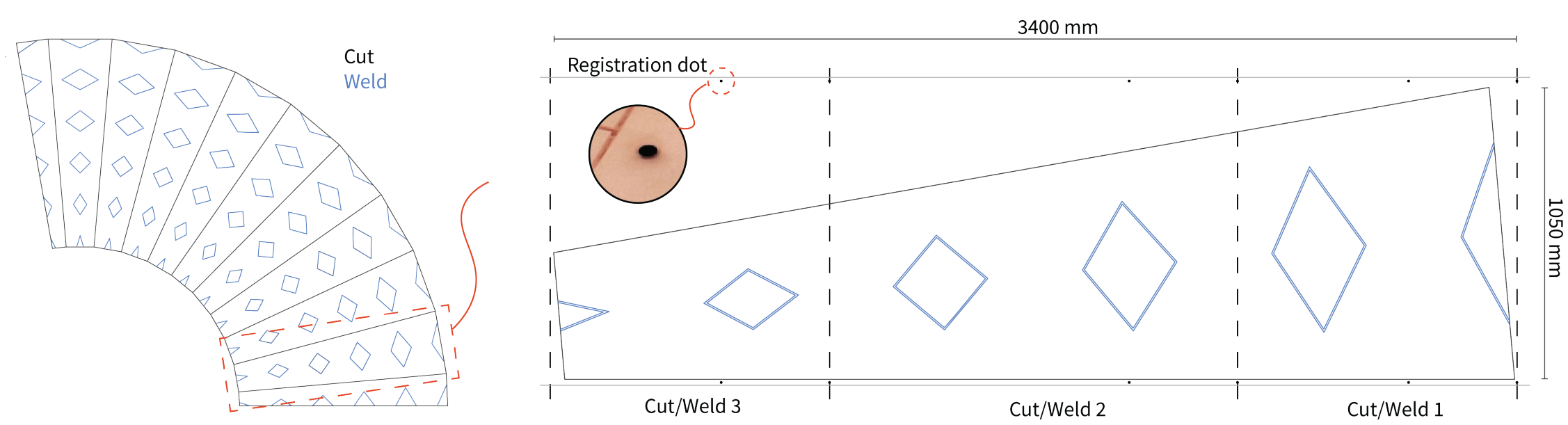

Fig. S2. Geometry of the large-scale inflatable. Schematic illustrating the details of the segments used to fabricate the meter-scale inflatable.

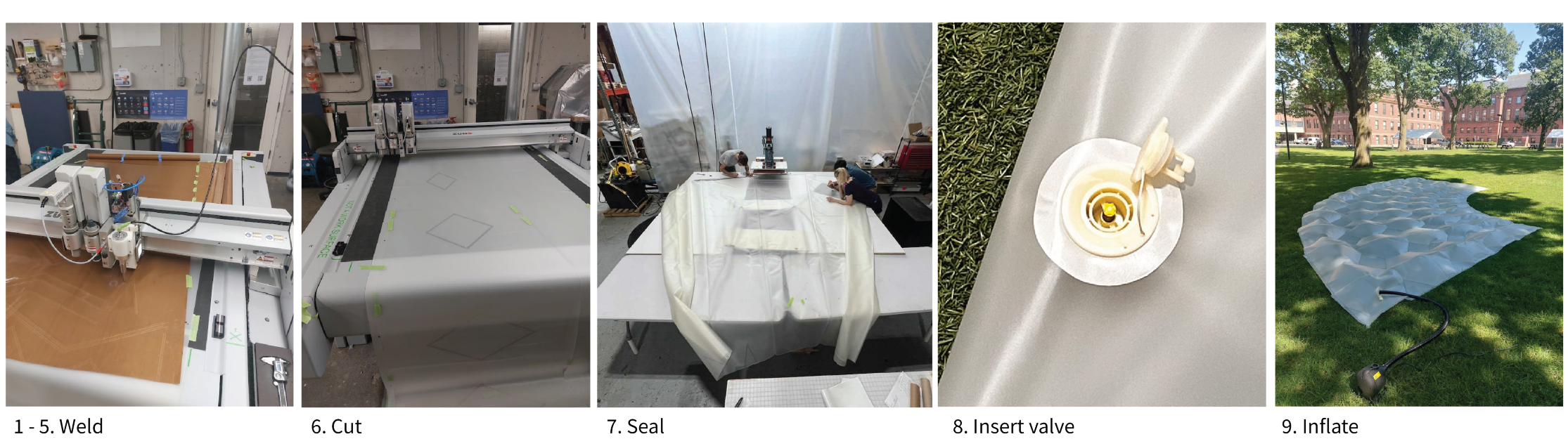

Fig. S3. Fabrication of the large scale inflatable. Snapshots of the steps required to fabricate our meter-scale inflatable.

S2. Bistable inflatable building block

In this section, we first describe the experimental and numerical procedures used to characterize the mechanical response of our inflatable building blocks. We then examine how geometric parameters influence their behavior. Finally, we explore the potential of leveraging the bistable nature of the proposed inflatable unit to create an on/off switch, demonstrating its functionality through the design of a lamp.

S2.1. Experimental Characterization.

S2.1.1. Inflation. To characterize the response of the fabricated inflatables under quasi-static inflation, we inflate the samples using both water and air as working fluids. To ensure repeatability, we fabricate and test two identical samples for each geometry.

Inflation with water To isolate the effect of sample geometry from fluid compressibility, we first determine the pressure–volume relationship by inflating the samples with water. As shown in Fig. S4, each sample is submerged in a water tank to eliminate gravitational effects. Water is injected at a controlled flow rate of 2 mL/min using a syringe pump (Pump 33DS, Harvard Apparatus), while the internal pressure is measured with a pressure sensor (MPXV7025DP, ±25 kPa range, NXP USA). Pressure and volume data are recorded synchronously over time. To ensure accurate measurements, all air is purged from the supply lines, and the pressure is calibrated to atmospheric pressure before each test.

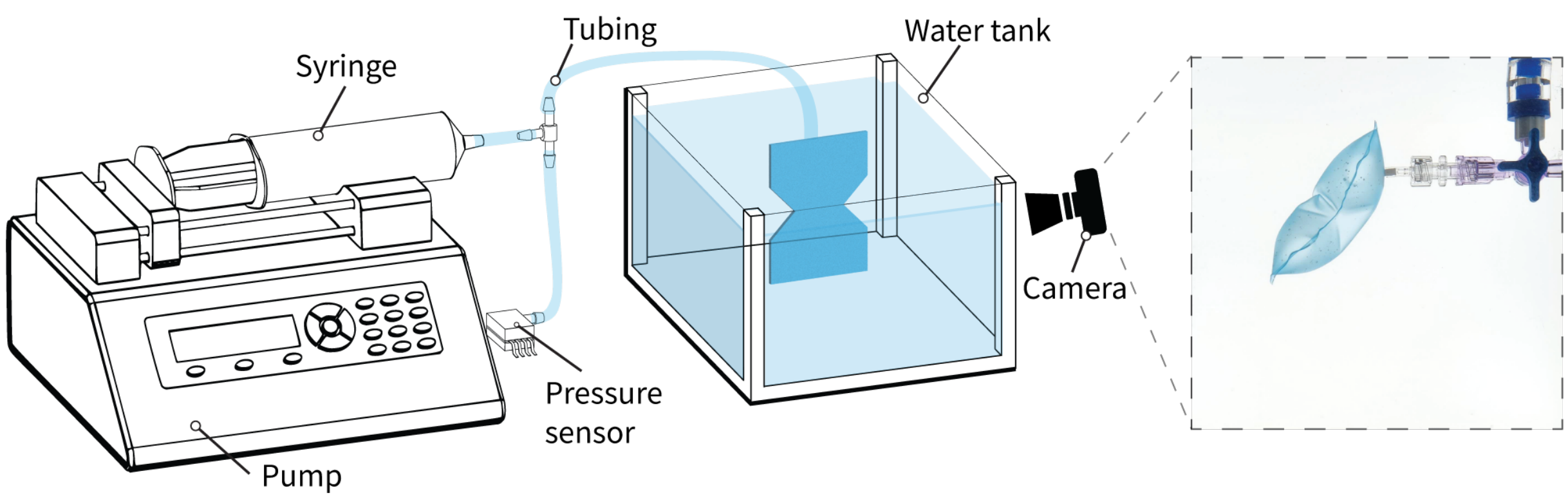

Fig. S4. Experimental setup for water inflation. Schematic of the test setup used to characterize the response of the samples when inflated with water. The photo on the right highlights the deformation triggered by crumple formation.

Inflation with air To investigate the influence of fluid compressibility on the response of the inflatables, we inflate the samples with air. For these tests, we use the same pump (33DS, Harvard67 Apparatus) and pressure sensors (MPXV7025DP, ±25 kPa range, NXP USA) as described earlier, injecting air into the samples at a rate of 1 mL/min. Note that the current volume of the system (V), which includes the volume of the inflatable cavity, syringe, and connecting tubes, at a given pressure p, can be expressed as:

$$V = V_0^{\rm sys} + \Delta V - \Delta V^{\rm syringe}, \qquad \text{[S1]}$$

where $V_0^{\rm sys}$ is the initial volume of the system, which includes the syringe and connecting tubes, since the initial volume of the inflatable is zero when it is deflated. Additionally, ΔV denotes the volume change of the inflatable, and $\Delta V^{\rm syringe}$ represents the volume injected by the syringe. We assume that the flow rate is sufficiently small to treat the system as isothermal, and that the air within the closed system behaves according to the ideal gas law. Thus, we have

$$p_0 V_0^{\rm sys} = pV, \qquad \text{[S2]}$$

which, when combined with Eq. (S1), gives

$$\Delta V = \Delta V^{\rm syringe} - \left(\frac{p - p_0}{p}\right) V_0^{\rm sys}, \qquad \text{[S3]}$$

where $p_0 \approx 101.3$ kPa is the initial pressure of the system (atmospheric pressure in our case).

In Fig. S5, we compare the pressure-volume curves measured for the inflatable shown in Fig. 1c of the main text, inflated with water (blue curve) and air (purple curve). The results show excellent agreement between the two. This suggests that by controlling the pressure during inflation, we can achieve equivalent behaviors using both water and air.

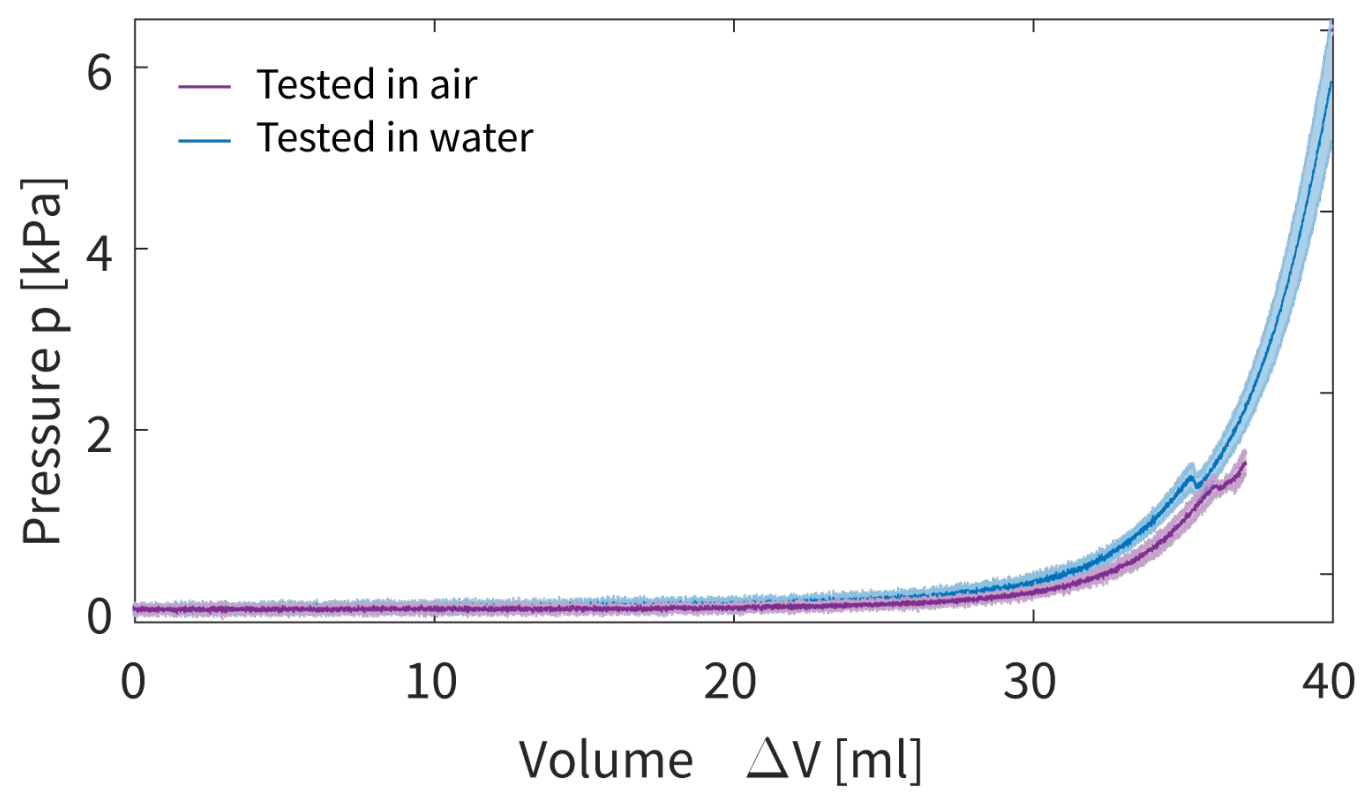

Fig. S5. Experimental pressure-volume curves. Pressure-volume curves for the inflatable shown in Fig. 1c of the main text, inflated with water (blue curve) and air (purple curve).

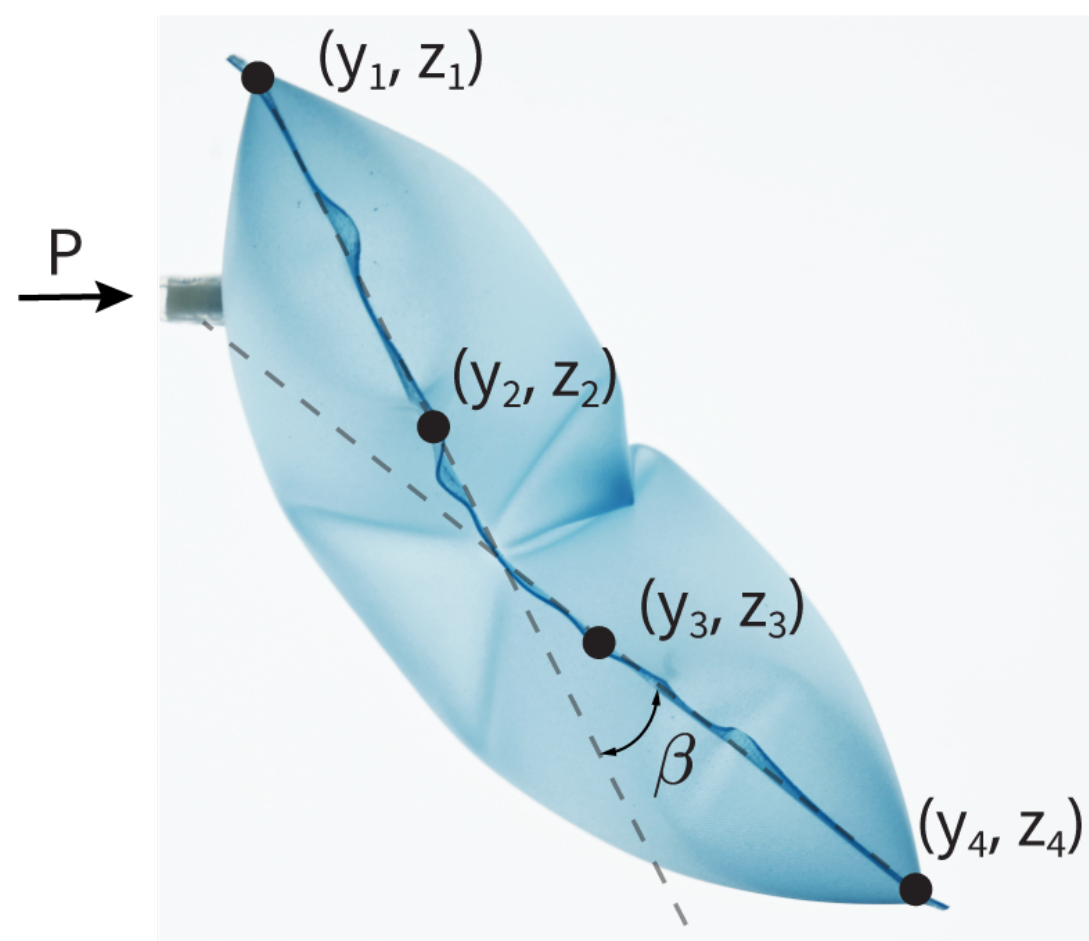

Fig. S6. Characterization of bending angle Images showing the markers tracked for calculating the bending angle (β).

S2.1.2. Characterization of bending angle. To quantify the bending angle introduced by crumpling, we track the positions of four markers arranged along one edge of the samples (Fig. S6). The positions of these markers are recorded during testing using a DSLR camera (Nikon Z6) at a frame rate of 30 fps, while the samples are inflated using a microfluidic flow controller (Fluigent, Flow EZ). the pressure is first increased from 0 to 7 kPa in increments of 0.5 kPa, and then from 7 to 25 kPa in increments of 1 kPa.

When the coordinates of the i-th marker in the deformed configuration are denoted with (y_i, z_i) , the bending angle β can be calculated as

$$\beta = \arctan\left|\frac{b_{3,4} - b_{1,2}}{1 + b_{1,2}b_{3,4}}\right|, \tag{S4}$$

with

$$b_{3,4} = \frac{z_4 - z_3}{y_4 - y_3}, \qquad b_{1,2} = \frac{z_2 - z_1}{y_2 - y_1}. \tag{S5}$$

S2.1.3. Characterization of the crumpling-induced hinges. To quantitatively characterize the bistable behavior of crumpling-induced hinges, we measure the restoring bending moment, M, as a function of the change in bending angle, $\Delta\beta$, under various internal pressures, p. These measurements are conducted using the setup shown in Fig. S7A. It consists of a pulley system made of laser-cut acrylic components that converts the linear displacement applied by an Instron (Model 5969) into rotational motion at the hinge. Note that two inextensible nylon strings are anchored at the edge of a wheel. To prevent contact between them, the wheel is designed with two layers of circular rings. The nylon string connected to the Instron is anchored to the inner layer at a radius of $R_i = 49$ mm, while the string attached to a counterweight mg is secured to the outer layer at a radius of $R_o = 50.5$ mm (Fig. S7C). A 20-gram counterweight ensures that the nylon string remains under tension throughout the loading process. Once inflated, the inflatable building block is secured onto the U-shaped clamp with bolts (Fig. S7B) and loaded by applying a displacement via the Instron.

Since the snap-through behavior of the inflatable structure is sensitive to variations in pressure and loading conditions, we minimize testing errors by fabricating three samples with identical geometry and testing each sample three times using the following protocol:

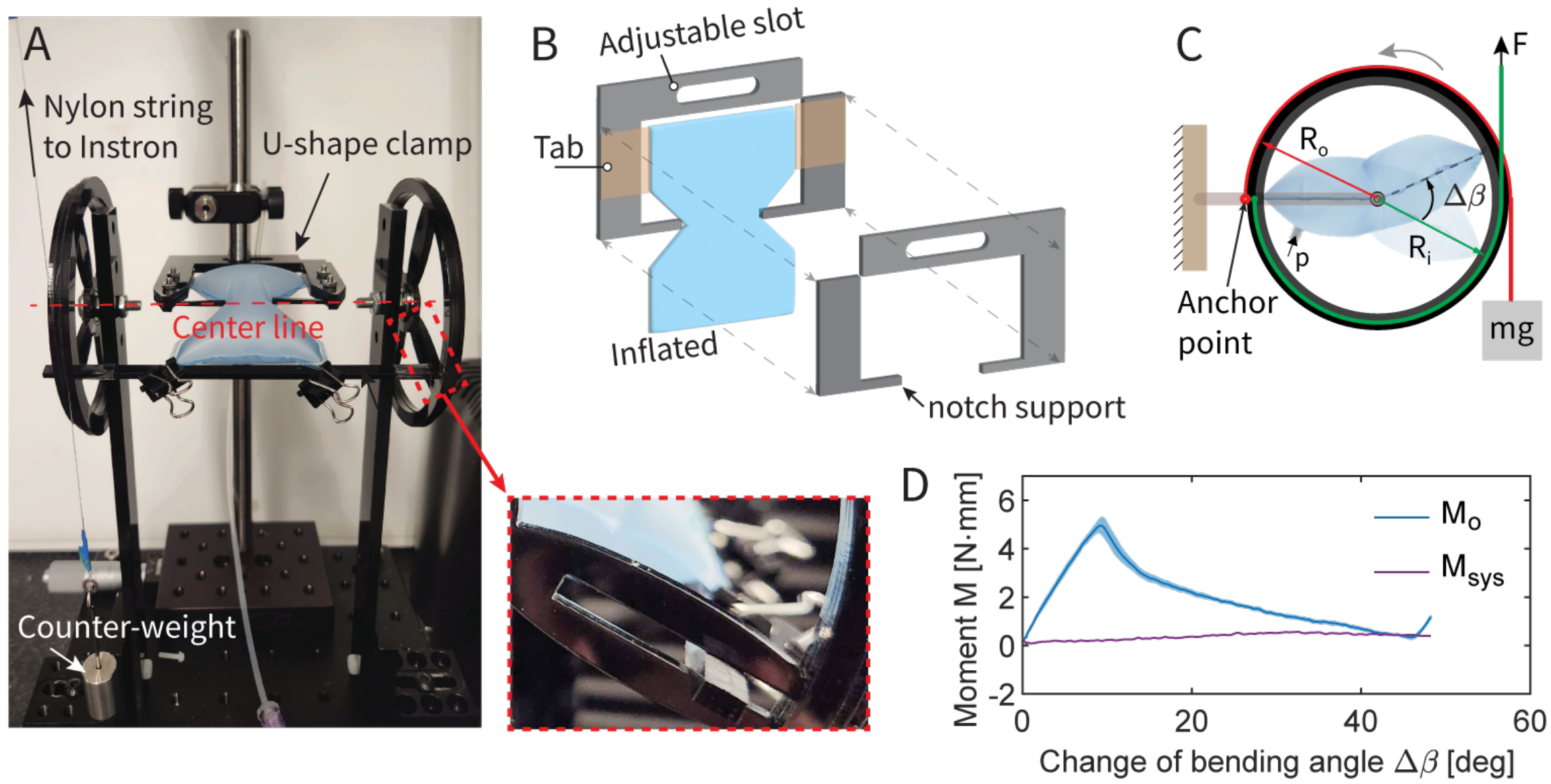

Fig. S7. Experimental setup for the characterization of the crumpling-induced hinges (A) Image showing the experimental setup. **(B)** Schematics showing how the inflatable building block is clamped. **(C)** Schematics showing the side view of the pulley. **(D)** Evolution of M_0 and M_{sys} as a function of $\Delta\beta$.

- **Step 1:** We inflate the sample to a pressure p and measure the width of the inflated sample.
- **Step 2:** We adjust the width of a laser-cut acrylic U-shaped clamp to match the measured width of the inflated sample and secure the sample by clamping the tabs connected to the side of the sample (Fig. S7B). We then align the center line of the sample (rotation center) with the rotational axis of the wheels and restrict any possible vertical displacement of the hinge (Fig. S7C).
- **Step 3:** We secure the opposite side of the sample to a sliding rod using two office paper clamps. The distance between the rod and the rotational center is adjusted to be exactly half of the inflated sample length. We then fix this distance to ensure that the sample rotates around its hinge.
- **Step 4:** We close the valve to ensure mass conservation during the tests.
- **Step 5:** We apply a quasi-static displacement to the Nylon string using the Instron 5969 moving at a velocity of 0.2 mm/s. The raw displacement data (u) is converted to change in bending angle of the hinge ($\Delta\beta$) using the geometric relationship,

  $$\Delta\beta = \frac{u}{R_i}, \tag{S6}$$

  where R_i is the moment arm shown in (Fig. S7C). The equivalent bending moment M_0 as a function of $\Delta\beta$ is then obtained as

  $$M_0(\Delta\beta) = F(\Delta\beta)R_i - mgR_o, \tag{S7}$$

  where $F(\Delta\beta)$ is the measured tensile force. Because the weights of the rod and paper clips are included in the measurement, their contribution must be subtracted. To account for the moment induced by additional weights and friction within the system M_{sys}, we measure $M_{sys}(\Delta\beta)$ as a function of the rotation angle $\Delta\beta$ (Fig. S7D). Finally, the bending moment contributed solely by the sample is obtained as

  $$M(\Delta\beta) = M_0(\Delta\beta) - M_{sys}(\Delta\beta). \tag{S8}$$

S2.1.4. Characterization of plasticity in TPU films. To evaluate whether the formation of crumples in the inflatable structures is an entirely elastic process, we characterized the mechanical response of TPU films with two different thicknesses ($t = 0.15$ mm and $t = 0.7$ mm). To quantify the residual strain associated with inelastic deformation, we conducted incremental loading–unloading tests on three identical strip specimens for each thickness. Each specimen had a length of 80 mm and a width of 10 mm. The tests were conducted using an Instron universal testing machine (Model 5969) equipped with a 500 N load cell and operated at a displacement rate of 0.2 mm/s.

Figures S8A and B show the corresponding stress–strain responses for three nominally identical strip specimens with thicknesses of $t = 0.15$ mm and $t = 0.7$ mm, respectively. During unloading, the stress decreased approximately linearly; however, the residual strain increased progressively with each loading cycle, indicating the accumulation of irreversible deformation. For strain levels up to approximately $\varepsilon = 0.05$, the residual strain remained small ($\varepsilon_{\mathrm{res}} < 0.002$), suggesting that the deformation

was largely recoverable. In contrast, loading to $\varepsilon = 0.15$ resulted in a substantially larger residual strain of approximately $\varepsilon_{res} \approx 0.008$, providing clear evidence of permanent deformation.

In addition, finite element simulations of the inflatable building block shown in Fig. S16 indicate that, during crumple formation, the material experiences localized stresses that exceed the yield stress, resulting in pronounced stress concentrations along the crumple lines. Consequently, upon deflation, residual crease lines remain visible on the TPU surface at the locations of the crumples, as shown in the inset of Fig. S8A, indicating that the crumpling process is not fully elastic. Importantly, however, this localized plastic deformation does not impair the bistable behavior of the structures presented in this work.

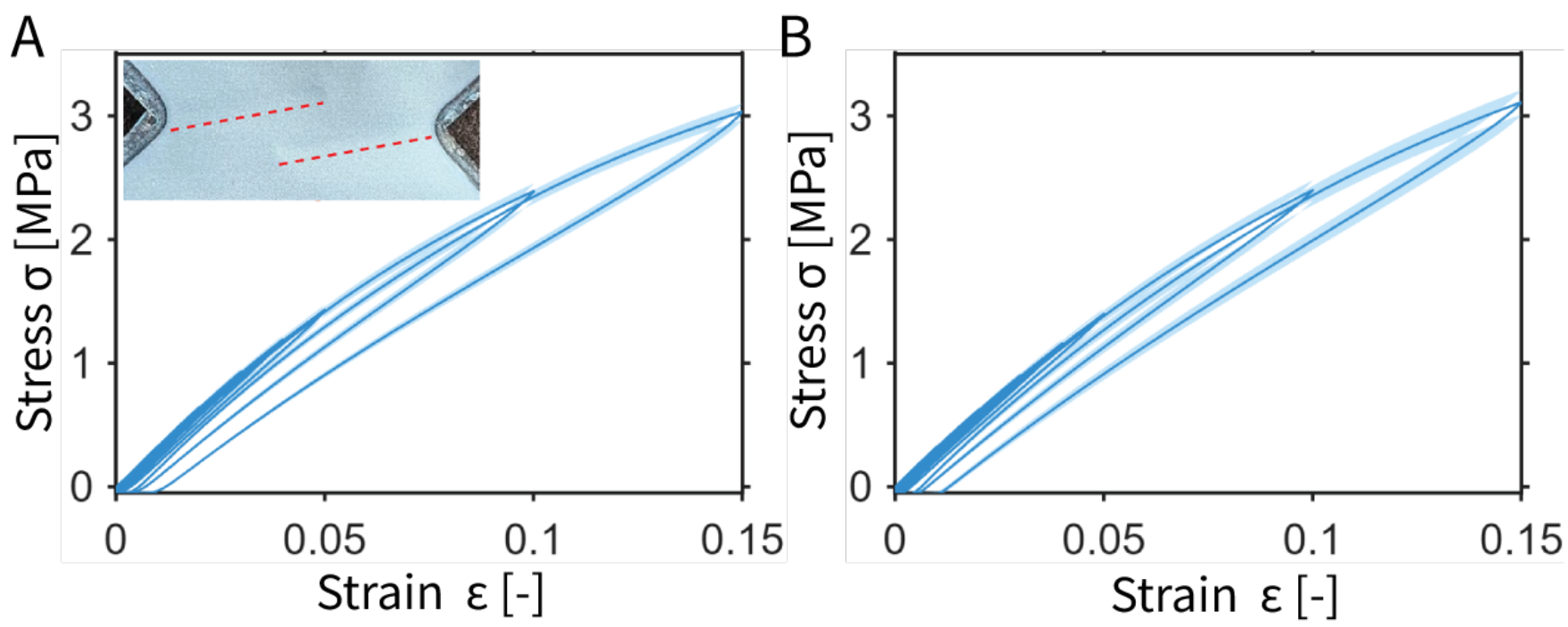

Fig. S8. Mechanical response of TPU sheets. Stress-strain relations for TPU films with thickness (A) $t = 0.15$ mm and (B) $t = 0.7$ mm under cyclic loading to successively higher strains (ε = 0.01, 0.02, 0.03, 0.04, 0.05, 0.1, 0.15). The inset shows the local plastic deformation in the TPU after deflation of the crumpled pouch, highlighted by the red dashed line.

S2.2. Effect of geometric parameters on mechanical response. The geometry of our inflatable building block is defined by four parameters: length (a), width (b), notch angle (θ), and spacing between two notches (s). These geometric features play a crucial role in how crumples develop upon inflation, thereby influencing the bistability of the structure. To explore the influence of geometry, we take the configuration shown in Fig. 1 of the main text as our baseline (with $a = 80$ mm, $b = 60$ mm, $s = 30$ mm, and $\theta = 90°$), and systematically vary one geometric parameter at a time while keeping the others fixed. In all cases, the samples are inflated at a constant pressure of 6 kPa.

We begin by varying the pouch length from $a = 60$ mm to 140 mm in 20 mm increments. Across all lengths tested, the crumple reliably extends the central width s, and each configuration exhibits bistable behavior, although producing different bending angles β at $p = 6$ kPa. As observed in Fig. S9A, β increases monotonically with the normalized length a/b.

We then vary the central width s from 15 mm to 35 mm in 5 mm increments, keeping all other parameters fixed. As shown in Fig. S9B, the bending angle β decreases monotonically with increasing normalized spacing s/b. For $s > 35$ mm, the crumple no longer spans the full distance between the notches, causing the building block to lose its bistable behavior and remain flat (i.e., $\beta = 0$). For these configurations, we also measured the bending moment as a function of bending angle at $p = 6$ kPa, as detailed in Section S2.1.3. The results indicate that the moment required to induce crumpling increases monotonically with s (Fig. S10A). Nevertheless, for all samples with $\beta \neq 0$, the moment eventually becomes negative, confirming the presence of bistability.

Next, we vary the notch angle θ from 30° to 150° in steps of 30°. As shown in Fig. S9C, the bending angle β decreases monotonically with increasing θ. At $\theta = 150°$, the crumple no longer bridges the notches, and the building block becomes monostable. As before, we measured the bending moment versus bending angle at $p = 6$ kPa. The results show that the required moment increases with θ (Fig. S10B), yet for all cases where $\beta \neq 0$, it eventually becomes negative, again confirming bistability.

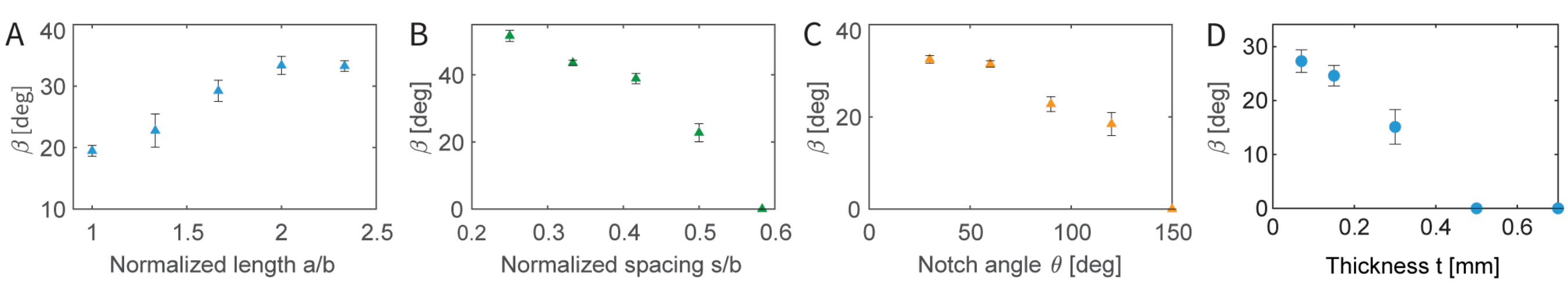

Fig. S9. Effect of geometric parameters on bending angle. Experimental measurements of the bending angle β at $p = 6$ kPa for building blocks with varying: (A) length a, (B) notch spacing s, (C) notch angle θ, and (D) thickness t

In addition to the building block geometry, the membrane thickness plays a key role in governing crumple formation and the resulting bistable behavior. To investigate this effect, we fabricated the reference geometry shown in Fig. 1 of the main text ($a = 80$ mm, $b = 60$ mm, $s = 30$ mm, $\theta = 90°$) using TPU membranes with five different thicknesses ($t = 0.07$, 0.15, 0.3,

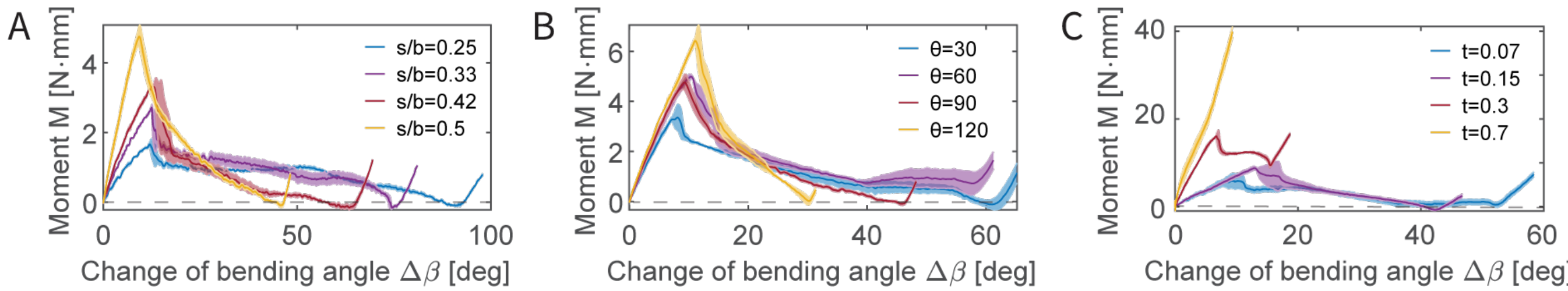

Fig. S10. Effect of geometric parameters on the mechanical response. Experimental measurements of bending moment M as a function of the change in bending angle $\Delta\beta$ for building blocks with varying: (A) notch spacing s, and (B) notch angle θ, and (C) thickness t.

0.5, and 0.7 mm). As shown in Fig. S9D, the bending angle β at $p = 6$ kPa decreases monotonically with increasing values of θ, and the samples with $t = 0.5$ and 0.7 mm remain flat. However, it is important to note that at $p = 6$ kPa, bistability is observed only in the thinnest membranes, with $t = 0.07$ mm and $t = 0.15$ mm (Fig. S10C). For $t = 0.3$ mm, the inflatable can snap but does not reliably maintain the snapped state. For $t = 0.5$ and 0.7 mm, the structure behaves like a pressurized beam, with no observable bending angle and bistable response.

Finally, we investigate the effect of scale on the response of the building block. Towards this end, we test samples in which all geometric parameters, including the out-of-plane thickness, are uniformly scaled. In particular, we consider the following four samples:

- scale factor $SF = 1$ (baseline): $a = 80$ mm, $b = 60$ mm, $s = 30$ mm, $t = 0.15$ mm and $\theta = 90^\circ$
- scale factor $SF = 2$: $a = 160$ mm, $b = 120$ mm, $s = 60$ mm, $t = 0.3$ mm and $\theta = 90^\circ$
- scale factor $SF = 3.3$: $a = 264$ mm, $b = 198$ mm, $s = 100$ mm, $t = 0.5$ mm and $\theta = 90^\circ$
- scale factor $SF = 4.7$: $a = 373$ mm, $b = 280$ mm, $s = 140$ mm, $t = 0.7$ mm and $\theta = 90^\circ$

For these samples, we first measure the bending angle β at $p = 6$ kPa. As shown in Fig. S11A, all four samples exhibit nearly identical bending angles, with $\beta \sim 25$ degrees. Next, we experimentally measure the moment M required to induce a change in the bending angle $\Delta\beta$ at $p = 6$ kPa. As expected, the results shown in Fig. S11B indicate that larger samples require a larger moment to induce snapping. However, when the measured moment is normalized by st^2, the curves collapse onto a single master curve, suggesting that

$$M \sim st^2. \quad \text{[S9]}$$

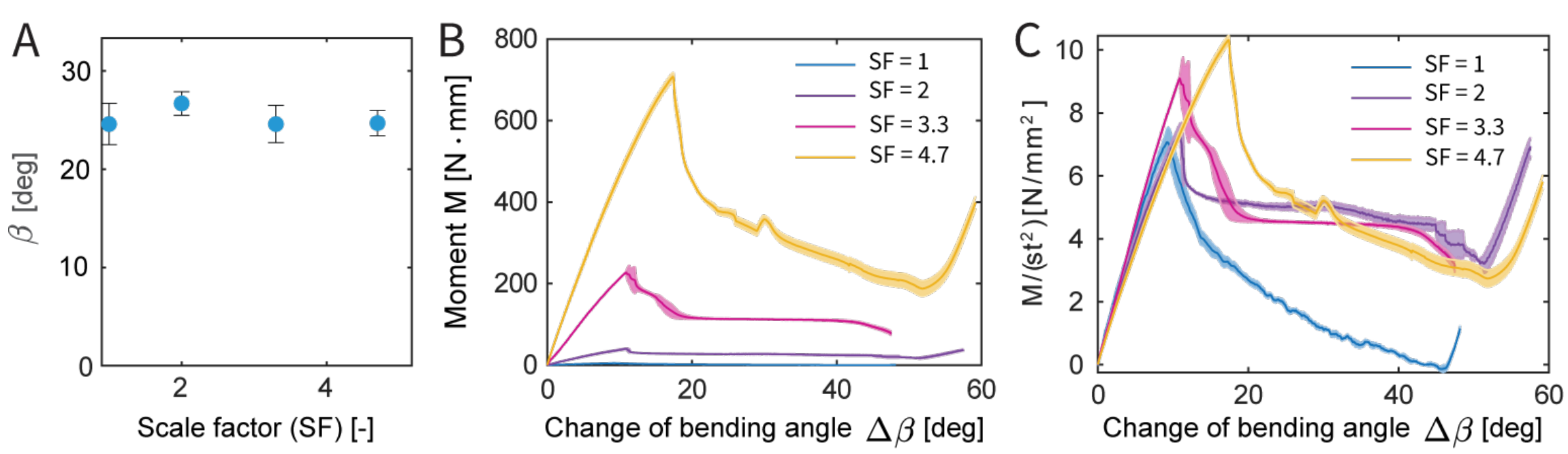

Fig. S11. Effect of scale on mechanical response. (A) Measured bending angle at $p = 6$ kPa vs scale factor. (B) Experimentally measured bending moment M as a function of change of bending angle $\Delta\beta$. (C) Experimentally measured bending moment M normalized by st^2 as a function of change of bending angle $\Delta\beta$.

Owing to the complex geometry of the building block considered here, a closed-form analytical expression for the bending moment M is generally not attainable. Nevertheless, analytical predictions exist for the critical bending moment required to trigger wrinkling in a bent cylindrical shell. For a material with Young's modulus E and Poisson's ratio ν, and a cylindrical shell of radius r and thickness t, the critical bending moment for the onset of wrinkling in an inflated shell under bending has been derived as (1)

$$M_{cr} = \pi p r^3 + \frac{\pi E r t^2}{\sqrt{3\,(1-\nu^2)}}. \quad \text{[S10]}$$

Furthermore, in the absence of internal pressure (i.e., for an unpressurized cylindrical shell), the critical bending moment reduces to (2)

$$M_{cr} = 0.987\,\frac{\pi E r t^2}{\sqrt{1-\nu^2}}. \quad \text{[S11]}$$

Although the geometry considered in this work is not cylindrical, Eqs. (S10) and (S11) both exhibit the scaling $M_{cr} \sim rt^2$, which is consistent with the scaling observed in our experiments.

S2.3. Effects of materials on mechanical response. To investigate the influence of the material used to fabricate the inflatable building block on its mechanical response, we fabricated the configuration shown in Fig. 1 of the main text ($a = 80$ mm, $b = 60$ mm, $s = 30$ mm, and $\theta = 90°$) out of three different materials.

- Building blocks fabricated with TPU films with a Young's modulus $E_{TPU} = 30.2$ MPa, Poisson's ratio $\nu_{TPU} = 0.35$ and thicknesses $t = 0.07$, 0.15, 0.3, 0.5 and 0.7 mm, all inflated at $p = 6$ kPa.
- A building block fabricated with TPU-coated Nylon fabric with a Young's modulus measured along three directions (0°, 45°, and 90°) as [322.5, 66.8, 203.1] MPa (the average Young's modulus $\bar{E}_{\text{Nylon}} = 197.5$ MPa), Poisson's ratio $\nu_{\text{Nylon}} = 0.4$, and thickness $t = 0.2$ mm.
- A building block fabricated with Mylar film with a Young's modulus $E_{Mylar} = 4.3$ GPa, Poisson's ratio $\nu_{Mylar} = 0.38$ and thickness $t = 0.15$ mm, inflated at $p = 6$ kPa. Note that inflating around 7 kPa will cause delamination of sealed lines.

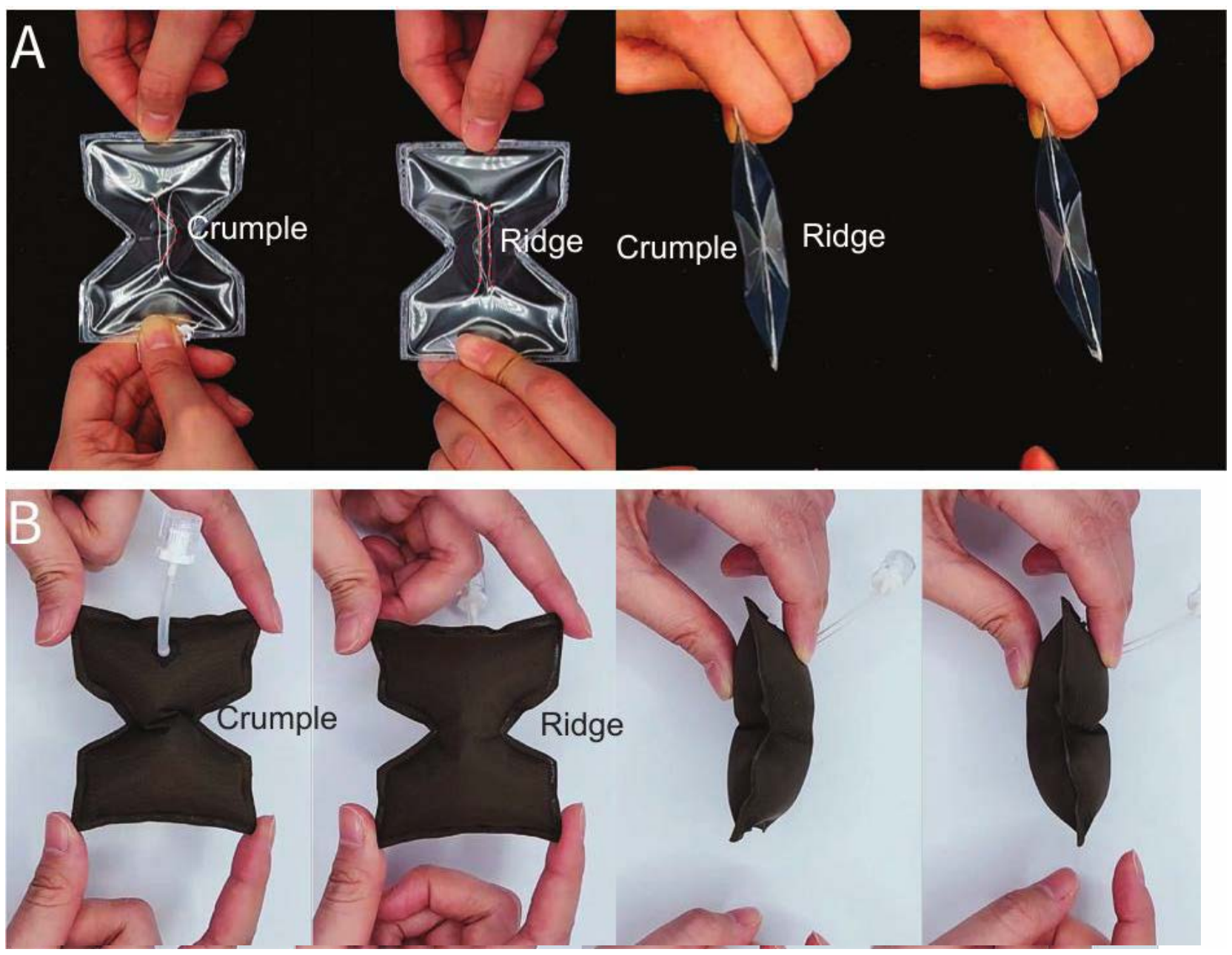

Fig. S12. Effect of materials on bistability. Images showing bistability of pouches fabricated with (A) Mylar film inflated at $p = 6$ kPa and (B) TPU-coated Nylon fabric inflated at $p = 17$ kPa

S2.4. Harnessing bistability to realize an on/off switch. In the main text, we focused on characterizing the bending angle β driven by crumple formation, as well as the forces and moments required to transition between the two stable states. Here, we examine how switching between these states affects the internal pressure of the inflatable and demonstrate how this pressure variation can be harnessed to create an on/off switch capable of powering a tabletop lamp.

We focus on an inflatable building block with dimensions $a = 160$ mm, $b = 120$ mm, $s = 60$ mm, $\theta = 90°$, and thickness $t = 0.3$ mm. The structure is inflated to an internal pressure of 6 kPa. To monitor pressure changes during switching, we use a pressure sensor (NXP MPXV7007DP) connected to an Arduino Uno. The inflatable is manually toggled between its two stable states six times at approximately 2.5-second intervals, and the pressure is recorded throughout the process (see Fig. S13A). Note that the size of the pouch is scaled up by a factor of two compared to the unit described in the main text. This was done to facilitate manual handling and to amplify the pressure differences between states, making them more discernible relative to the transient pressure spikes associated with switching. The recorded pressure response is shown in Fig. S13A (orange curve). Each transition between states is marked by a sharp pressure increase of approximately 0.6 kPa. Between these spikes, we observe that the pressure stays approximately at the same pressure with an average pressure difference of 0.04 ± 0.02 kPa between the two states. This discrepancy may arise from slight variations in the crumple patterns associated the two stable states.

This shows that the internal pressure cannot distinguish the state of the pouch, as they are symmetric. However, this changes when an asymmetry in the pouch is introduced by varying the thickness of the membrane. We create a new inflatable block

where the top membrane has a thickness of $t_1 = 0.3$ mm, while the bottom layer is thinner, with $t_2 = 0.15$ mm. This asymmetry introduces a strong bias for crumple formation on the thinner side, causing the structure to preferentially bend downward. Switching the inflatable to the upward configuration, where the crumple forms in the thicker top layer, is energetically less favorable, resulting in a pronounced asymmetry between the two states. This is now also reflected in the internal pressure of the pouch. First, we notice that the peak pressure for switching downward is approximately 0.4 kPa, whereas switching upward results in a peak of about 0.6 kPa (Fig. S13B - blue line), showing that it is easier to switch down. Moreover, the internal pressures associated with the two stable states are different. We observe a consistent pressure difference of 0.06 ± 0.01 kPa between the two states, with the configuration having the crumple in the thinner membrane exhibiting the higher pressure.

We leverage the feature of a bistable inflatable building block with two stable configurations, each associated with a distinct internal pressure, to create a pressure-based on/off switch. Previous studies have explored multistable inflatables as switches, primarily due to the haptic feedback generated by the snap-through transition (3). However, detecting state changes typically requires embedding electronic components within the inflatable, which compromises its softness and translucency. In contrast, our approach allows all electronic components to remain external to the inflatable structure, because it uses internal pressure as the sensing mechanism. As shown in Fig. S13B-C, we demonstrate this concept through the realization of a table-top lamp. The inflatable building block is pressurized to a baseline of $p_{\text{lamp}} \approx 6$ kPa. The control system is programmed to switch the light on when the pressure falls within the range $p < p_{\text{lamp}} \pm 0.03$ kPa, and off when it lies outside this range. As a result, the light turns on when the pouch is in the upward (high-pressure) state and turns off when it returns to the downward (low-pressure) state (Fig. S13C).

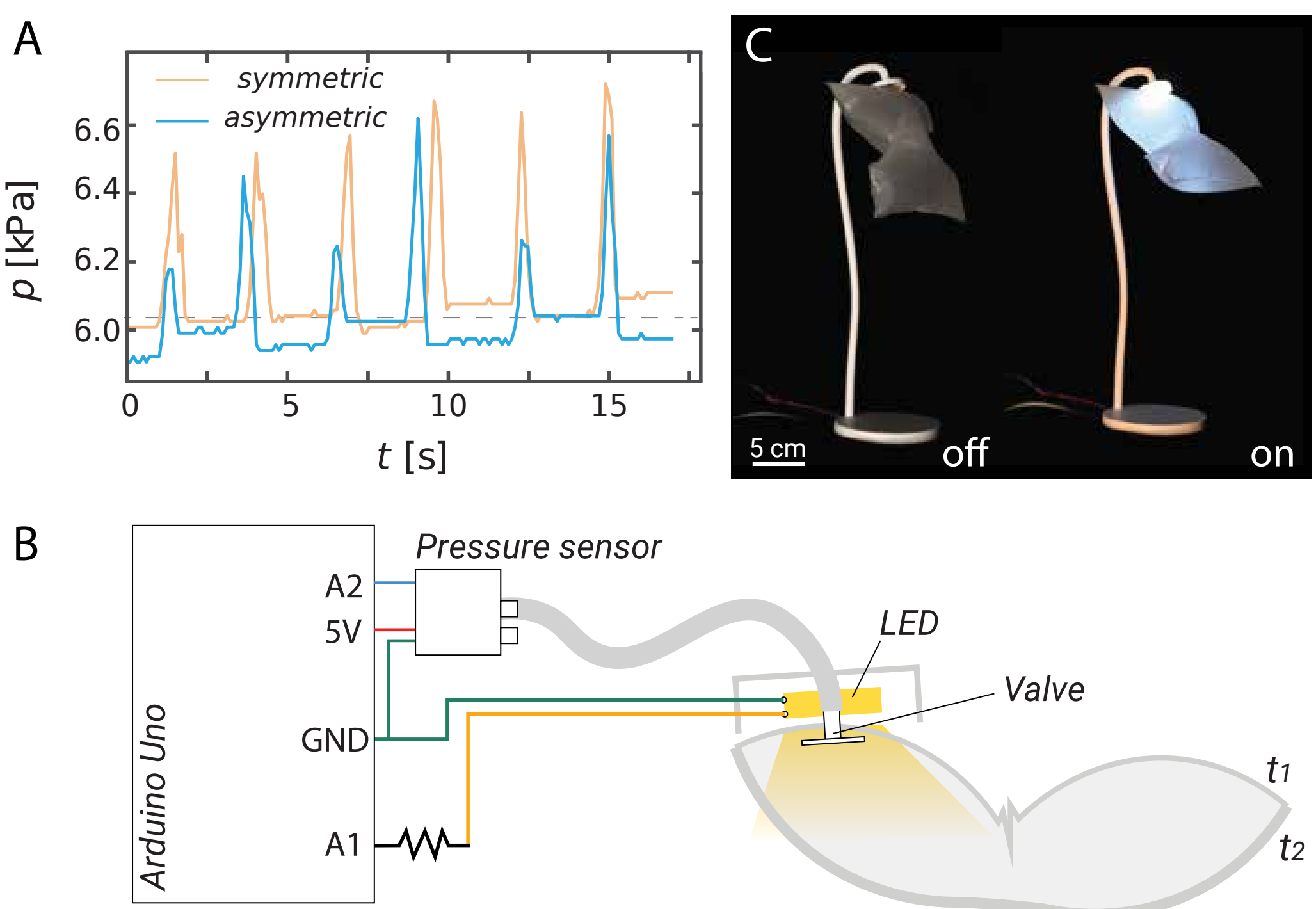

Fig. S13. On/off switch. a, Recorded pressure as the inflatable building block with membranes of identical (orange line) and different (blue line) thicknesses is manually toggled between its two stable states six times. **b**, Schematic of the tabletop lamp setup. **c**, Snapshots of the tabletop lamp with the inflatable building block in its upward (high-pressure, "on") state and downward (low-pressure, "off") state.

S2.5. Finite Element Simulations. In this Section, we describe the Finite Element (FE) simulations conducted to characterize the formation of crumpling in the inflatable building block during inflation. To accurately predict the behavior of an inflatable building block upon inflation, we conducted FE simulations using the commercial code Abaqus (Abaqus/CAE 2021, Dassault Systèmes).

Geometric model and discretization. The geometry of the inflatable building block used in the simulation corresponds to the fabricated unit used in experiments. It consists of two sheets with dimensions 79 mm × 60 mm, slightly adjusted from the nominal design to match the actual manufactured inflatable building block. The nominal thickness of the sheets is 0.15 mm. We discretize the model using four-node reduced-integration shell elements (S4R in Abaqus) with a global seed size of 1 mm, resulting in 4864 elements for the complete model (Fig. S14a). These elements account for finite membrane strains and large rotations, employing default hourglass control to prevent spurious modes.

Material model. The stress-strain behavior of the thermoplastic polyurethane (TPU) material used to fabricate the inflatable building block is captured using a first-order Ogden hyperelastic model. The strain energy density function is given by

$$\Psi = \frac{2\mu_1}{\alpha_1^2}\left(\bar{\lambda}_1^{\alpha_1} + \bar{\lambda}_2^{\alpha_1} + \bar{\lambda}_3^{\alpha_1} - 3\right) + \frac{1}{D_1}\left(J^{el} - 1\right)^2, \qquad \text{[S12]}$$

where $\bar{\lambda}_i = J^{(1/3)}\lambda_i$ are the deviatoric stretches with λ_i being the principal stretches and J being the total volume ratio. Furthermore, J^{el} represents the elastic volume ratio, while μ_1, α_1 and D_1 are the material parameters obtained by curve fitting, where μ_1 sets the overall shear stiffness of the material, α_1 controls the nonlinearity and tension–compression asymmetry of the stress–strain curve, and D_1 governs volumetric stiffness—penalizing changes in volume to enforce near-incompressibility. The first term of the strain energy accounts for the isochoric (volume-preserving) response, while the second term penalizes volumetric changes. We fit the model parameters to uniaxial tensile test data obtained experimentally from rectangular sheet samples with dimensions of 150 mm width and 75 mm height (Fig. S14A and B). We find that the response of TPU is best captured with: $\mu_1 = 11.2, \text{MPa}$, $\alpha_1 = -10$, and $D_1 = 0.06$. These parameters correspond to the following material properties: initial shear modulus $\mu_0 = 11.2, \text{MPa}$, initial bulk modulus $K_0 = 33.33, \text{MPa}$, initial Young's modulus $E = 30.22, \text{MPa}$ and Poisson's ratio $\nu = 0.3489$. Additionally, we set the material density to $1.23 \times 10^{-9}, \text{t/mm}^3$ and apply Rayleigh damping with $\alpha = 15\text{s}^{-1}$. Fig. S14B shows the comparison between the experimental stress-strain data and the fitted Ogden model prediction.

Boundary conditions. We constrain all translational degrees of freedom along the top edge of the pouch, effectively fixing it in space (Fig. S14C). Additionally, to account for the influence of the connecting tube required for inflation, we apply further constraints to a circular area on one of the two sheets. This area, corresponding to the tube's cross-section, is restrained in the x and y directions (in-plane displacements).

Loading conditions. To simulate the inflation of the pouch, we define a fluid cavity within the pouch and control its volume through a variable temperature field. We linearly increase the cavity volume to simulate a volume-controlled process, mimicking the experimental inflation process. By using volume control rather than pressure control, we can capture any instabilities or sudden changes in the pouch's behavior during inflation.

Analysis. We perform dynamic implicit analyzes with an inflation time of 20 s to capture inertial effects and large deformations, accounting for geometric nonlinearities to capture the complex deformation patterns observed experimentally.

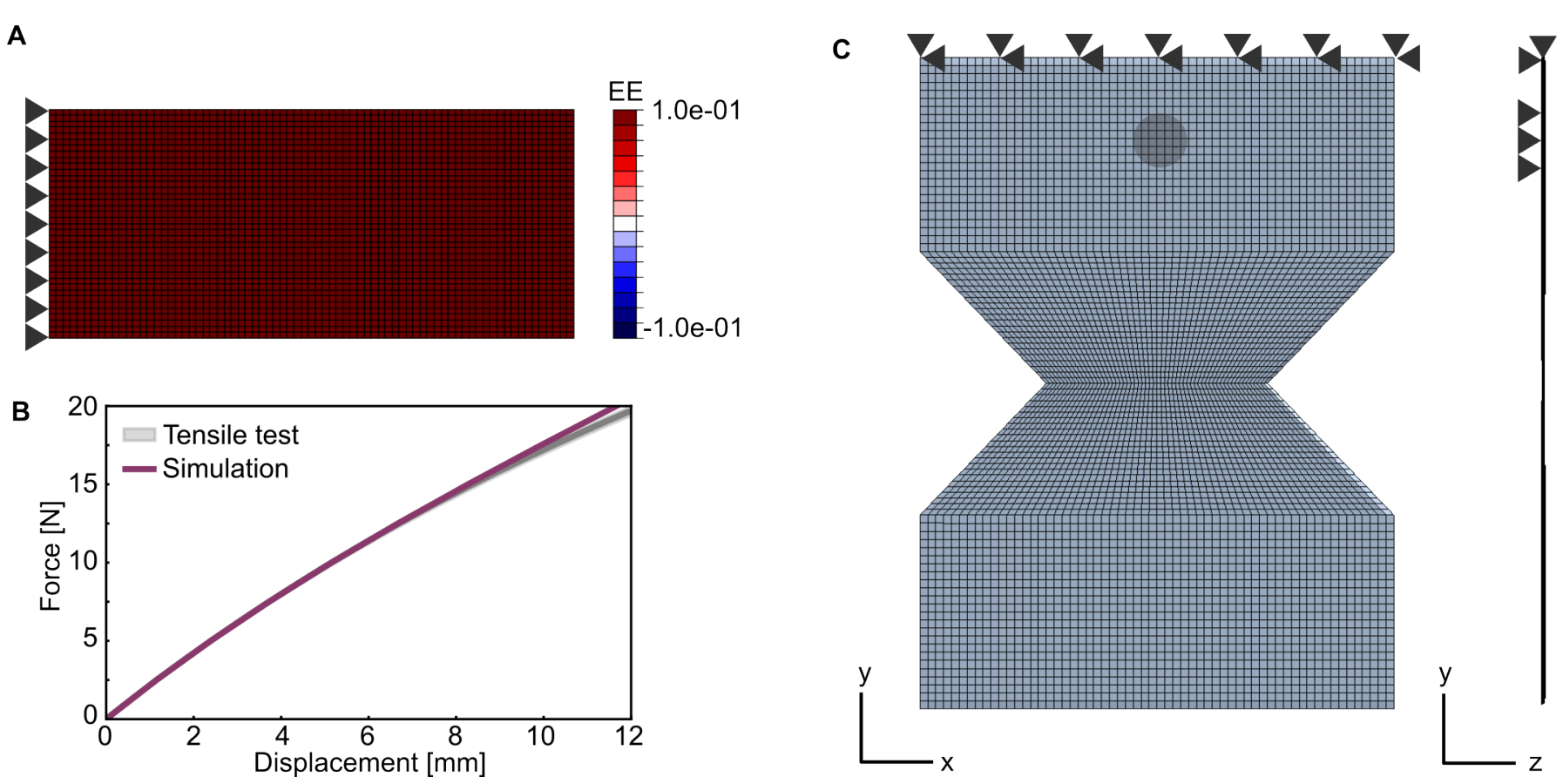

Fig. S14. Finite element model and material validation. (A) Rectangular sheet for the simulated tensile test with resulting elastic strain distribution (EE). (B) Comparison between the experimentally measured force-displacement response (gray) and the simulated response (purple curve) with the fitted hyperelastic Ogden model. (C) Finite element model of the inflatable unit.

Twisting instability. The dynamic implicit analysis of a geometrically perfect system (without imperfections) produces a pressure-volume relationship that closely tracks our experimental measurements throughout the inflation process. At a volume of $V \approx 21.5$ml, the pressure-volume curve exhibits a distinct drop as the displacement of the bottom corners of the structure diverge, indicated by ① in Fig. S15A and Fig. S15B. This marks the critical point (bifurcation) where a symmetry-breaking bifurcation occurs: the structure transitions from an axisymmetric shape into a twisted configuration, breaking symmetry around the vertical (y-)axis. This symmetry break is captured in the three sequential deformation states in Fig. S15C with the contour plot of the in-plane principal stress and the cross-sectional views in Fig. S15D.

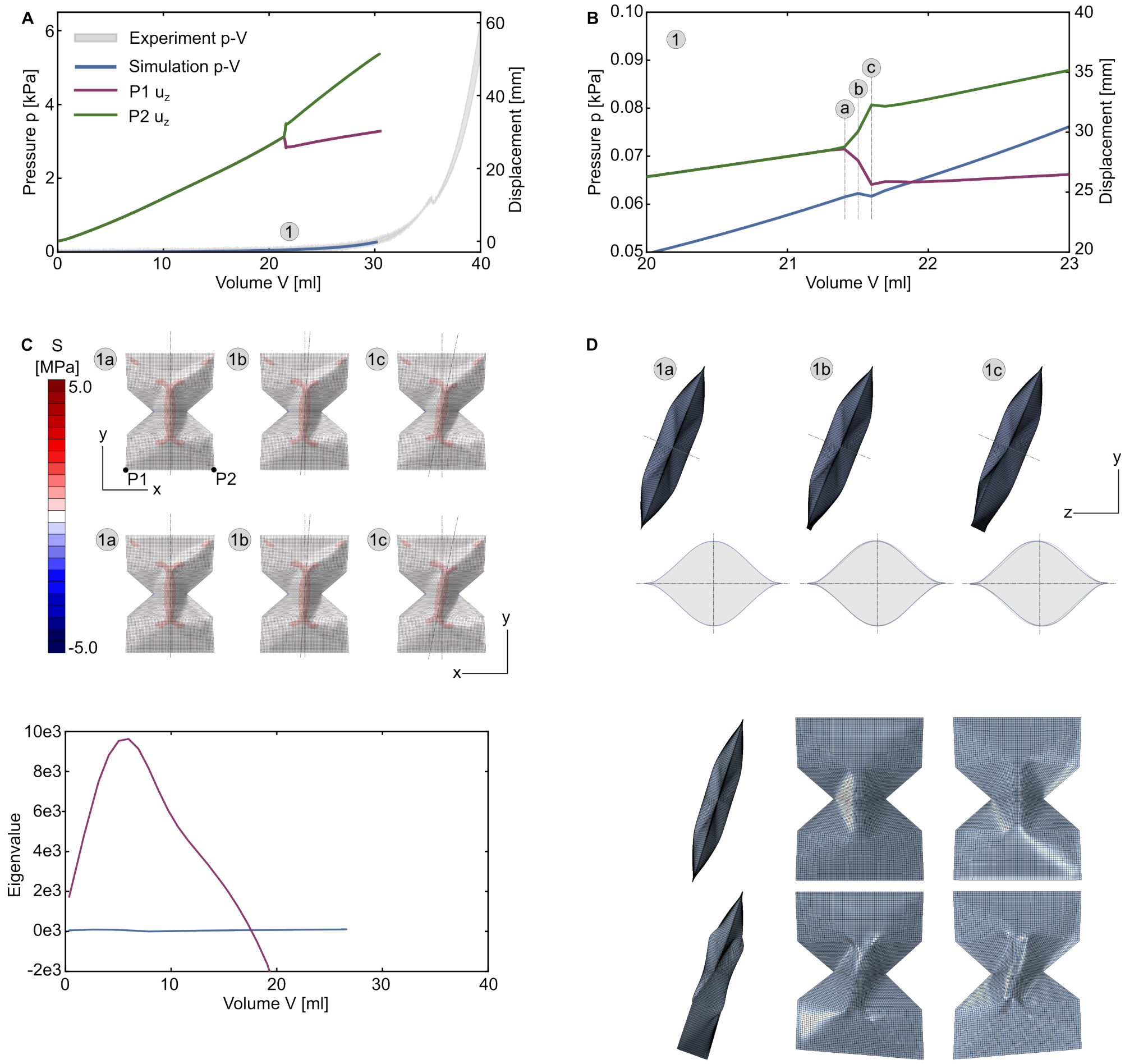

Fig. S15. Twisting instability in the inflatable unit. (A) Pressure–volume curves for both simulation (blue line) and experiment (gray shading), along with the z-displacement u_z of the bottom corners (P1 and P2). A clear drop in pressure appears at $V \approx 21.5$ml, coinciding with the rapid divergence of corner displacements. The marker ① indicates the onset of this transition. (B) Zoomed-in view around the bifurcation point ① with labeled deformation states a, b and c, corresponding to the three states traced in panels C and D. (C) Deformed geometries at the three states a, b and c, overlaid with contour plots of the maximum in-plane principal stress (color scale in MPa) in front and back view of the pouch. (D) Corresponding mid-height cross-sections for the deformation states a, b and c, illustrating the loss of vertical-axis symmetry.

Crumpling instability. To isolate the crumpling phenomenon, we introduce the twisting mode as a geometric imperfection (maximum out-of-plane displacement of 0.23 mm) to guide the structure into its twisted state. This imperfection eliminates the abrupt pressure drop in the pressure-volume curve at a volume of $V \approx 21.5$ml, resulting in a smooth transition into the twisted configuration, evidenced by the gradual displacement of the bottom corners (Fig. S16A). The simulated pressure-volume curve again shows good agreement with experimental measurements, and the predicted bending angles align precisely with experimental data in regions where optical tracking is challenging during rapid transitions (Fig. S16E). The crumpling instability emerges at $V \approx 35.9$ml through a two-step process, indicated by the marker ②. Initially, we observe a significant change in the bending angle coinciding with a divergence in strain energy between the front (crumpling) and back (ridge) sheets (Fig. S16C and Fig. S16D). These effects intensify at $V = 36.6ml$, marked by larger changes in both the bending angle and strain energy difference, while the bottom corners converge, partially resolving the twisted configuration as illustrated by the displacement u_z of the corner points P1 and P2 in Fig. S16B. This instability breaks symmetry about the horizontal axis, initiating the structure's bistable behavior as shown in the deformation states in Fig. S16F, Fig. S16G and Fig. S16H.

This analysis not only proves the relevance of instabilities in our system but also clearly identifies the unstable modes. It provides crucial insights into the sequential instability mechanisms - from twisting to crumpling - that govern the pouch's behavior during inflation.

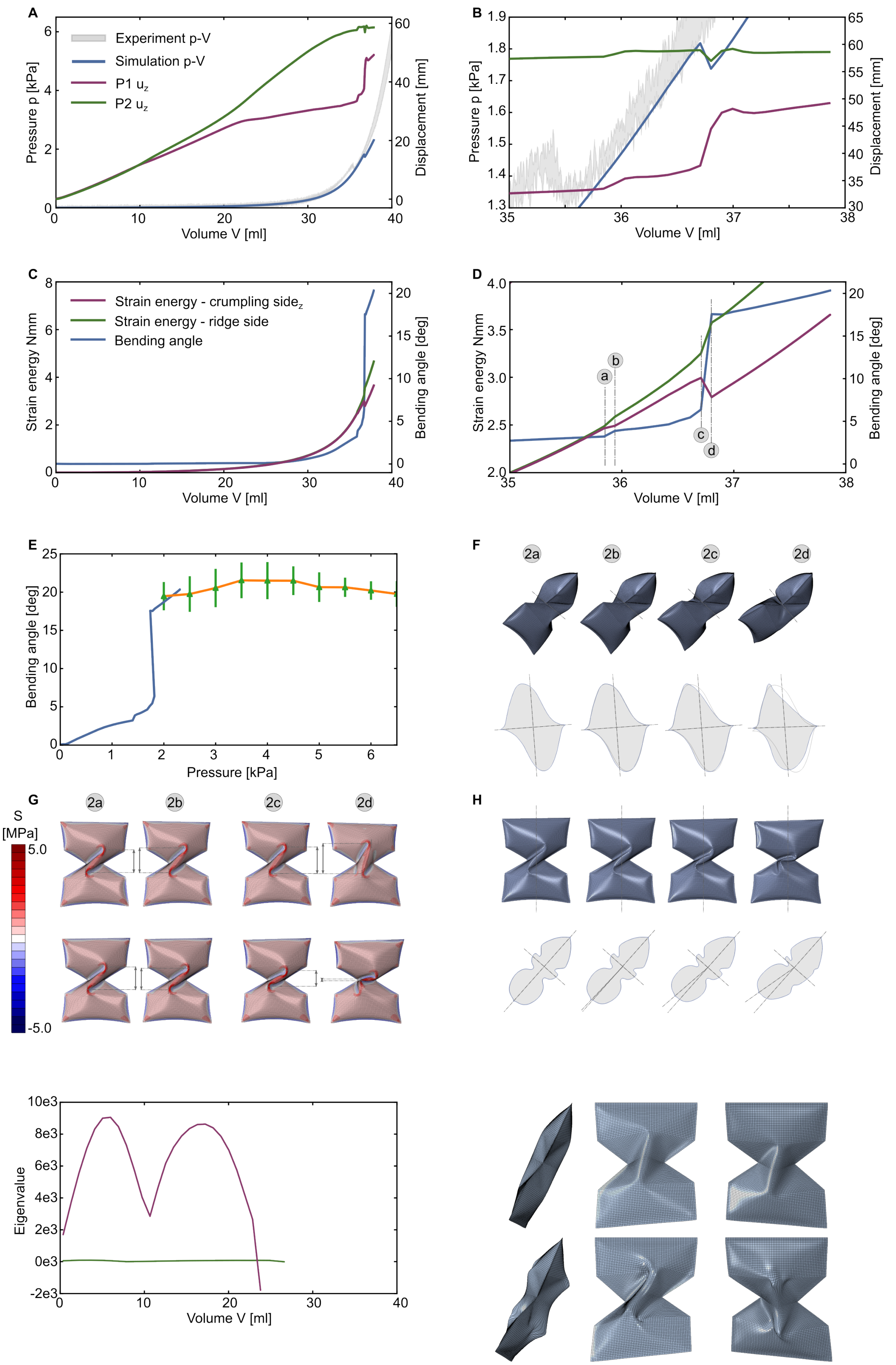

Fig. S16. Crumpling instability following twisted state. (A) Pressure–volume relationship from simulation (blue) and experiment (gray), showing a smooth transition into the twisted state after introducing a geometric imperfection based on the twisting mode. (B) Zoomed-in view around the crumpling onset. (C) The strain energy evolution reveals divergence between the crumpling and ridge sides at $V \approx 35.9 - 36.6$ml, accompanied by changes in bending angle. (D) Zoomed-in view of (C) around the crumpling transition, with indicated deformation states a,b and c visualized in subsequent panels. (E) Bending angle as a function of pressure. (F) Sequential deformed configurations at states a, b and c, demonstrating the progression from twisted to crumpled state, breaking symmetry about the horizontal axis. (G) Corresponding contour plots of maximum in-plane principal stress, highlighting stress localization in the crumpling region. (H) Mid-height cross-sectional views of the deformed geometries, demonstrating the symmetry-breaking transition about the horizontal axis.

S3. Multistable inflatable tessellations

In this section, we present the experimental characterization of curvature in our 1D and 2D multistable inflatable tessellations, along with design details of the configurations investigated in this study. We also describe the experimental setup for the drop tests, as well as the reduced-order model used to predict the drop height required to trigger snapping for a given object mass.

S3.1. Characterization of curvature. The curvature of 1D tessellations is measured using image processing techniques. Inflated samples are placed on a flat surface and photographed with a DSLR camera (Nikon Z6) positioned 1 meter away, focused on the center of the inflatable. The pixel coordinates corresponding to the midpoints of each hinge are manually selected, and a custom MATLAB script (MathWorks, Inc.) is used to fit a circle through these points (Fig. S17A). Curvature is then computed as $\kappa = 1/R$, where R is the radius of the fitted circle. All samples are inflated to 6 kPa prior to measurement.

To characterize the principal curvatures (κ_1 and κ_2) of 2D tessellations, we scan the inflated samples using a 3D scanner (Artec Eva) and process the data in CAD software (Rhino – Rhinoceros 3D). As shown in Fig. S17B, the 3D model is sliced along the principal directions (1–3 and 2–3 planes) using the MeshSplit command in Rhino. Pixel coordinates corresponding to the midpoints of each hinge on these cross-sections are manually extracted, and the curvature in each direction is calculated by fitting a circle through the selected points using the same MATLAB code. The principal curvatures are then given by $\kappa_1 = 1/R_1$ and $\kappa_2 = 1/R_2$, where R_1 and R_2 are the radii of the fitted circle in their respective principal planes.

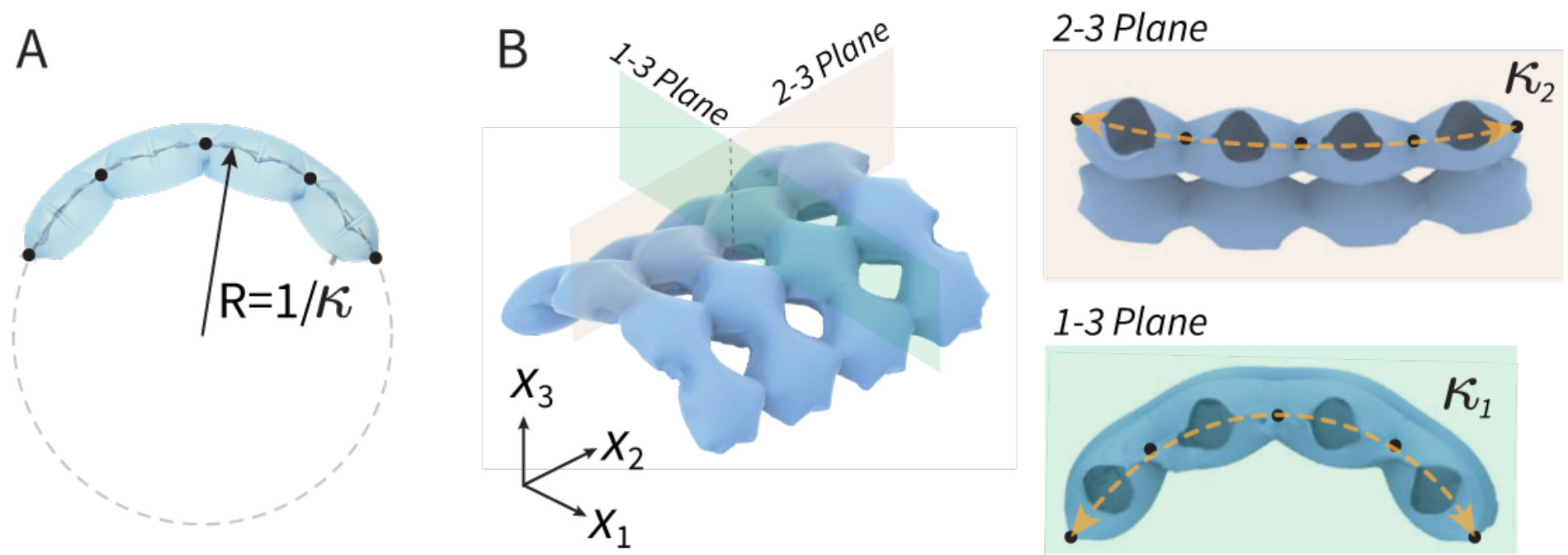

Fig. S17. Characterization of curvature (A) Curvature is determined by fitting a circle through the pixels corresponding to the midpoint of each hinge. **(B)** 3D models obtained using a 3D scanner showing the planes 1-3 and 2-3.

S3.2. Design details for 2D multistable tessellations. In Figs. S19–S21, we present the design details of the 2D tessellations shown in Fig. 3 of the main text, along with additional images of their stable configurations.

The design intuition for the tessellated multi-stable structures presented in this work is based on an origami-inspired approximation, in which the inflatable is modeled as a collection of rigid panels (regions without crumples) connected by hinges corresponding to the crumpled regions. This design process is illustrated in Fig. S18 for two different origami patterns.

We begin by defining a flat folding pattern that transforms into the desired three-dimensional shape. At each vertex of this pattern, a polygonal non-inflating region is placed, with its corners aligned along the fold lines. The geometry of these polygons can be tuned to set the spacing s required for the formation of a stable crumple and to achieve a target bending angle, which can be estimated using the data for the inflatable building block reported in Fig. S8.

While the main text focuses on diamond-shaped non-inflating regions that generate four-vertex folds, Fig. S18b demonstrates a six-vertex design that produces a honeycomb arrangement of hexagonal non-inflating regions. Upon inflation, crumples form along the prescribed directions, confirming that this geometric design approach effectively guides the resulting folded configuration.

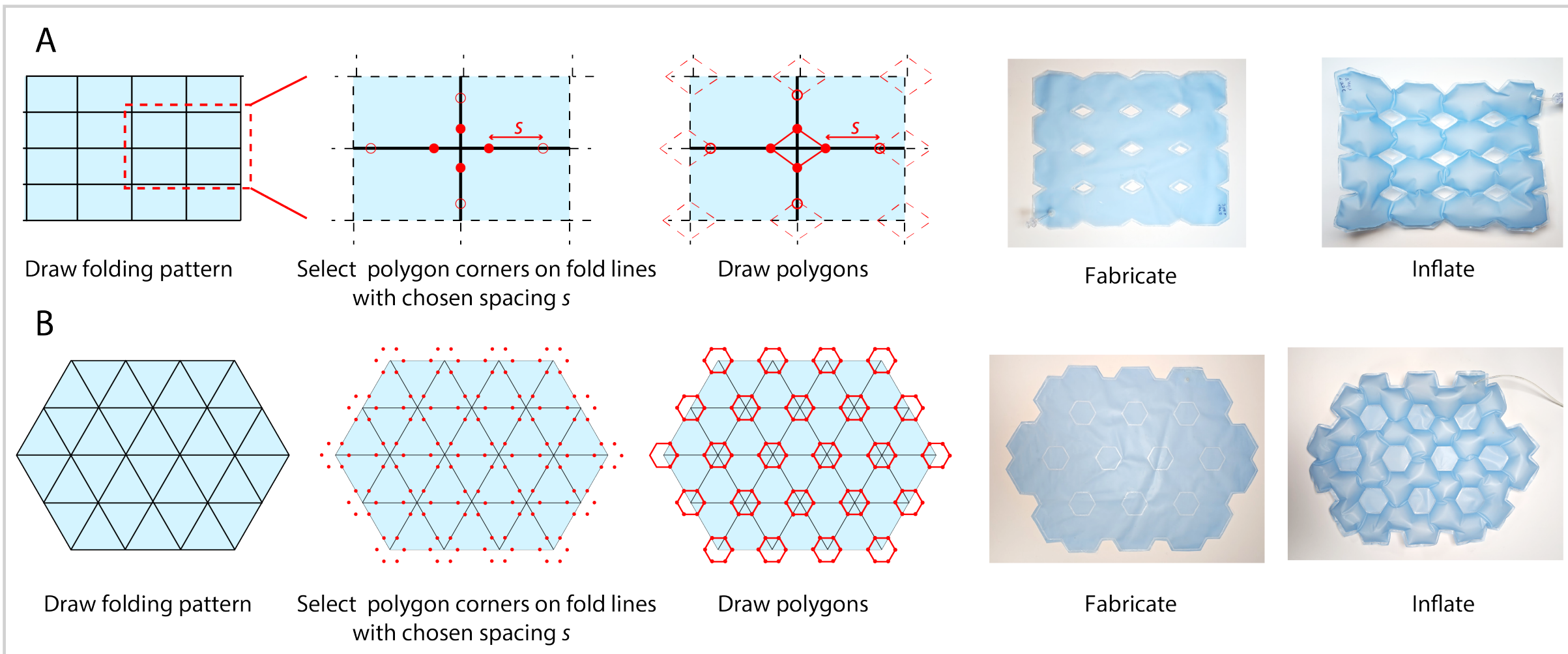

Fig. S18. Design guidelines for tessellated multi-stable structures. From left to right: key steps in the design process for tessellated multi-stable structures, along with snapshots of the fabricated inflatable before and after inflation, for patterns with (A) four-fold vertices and (B) six-fold vertices.

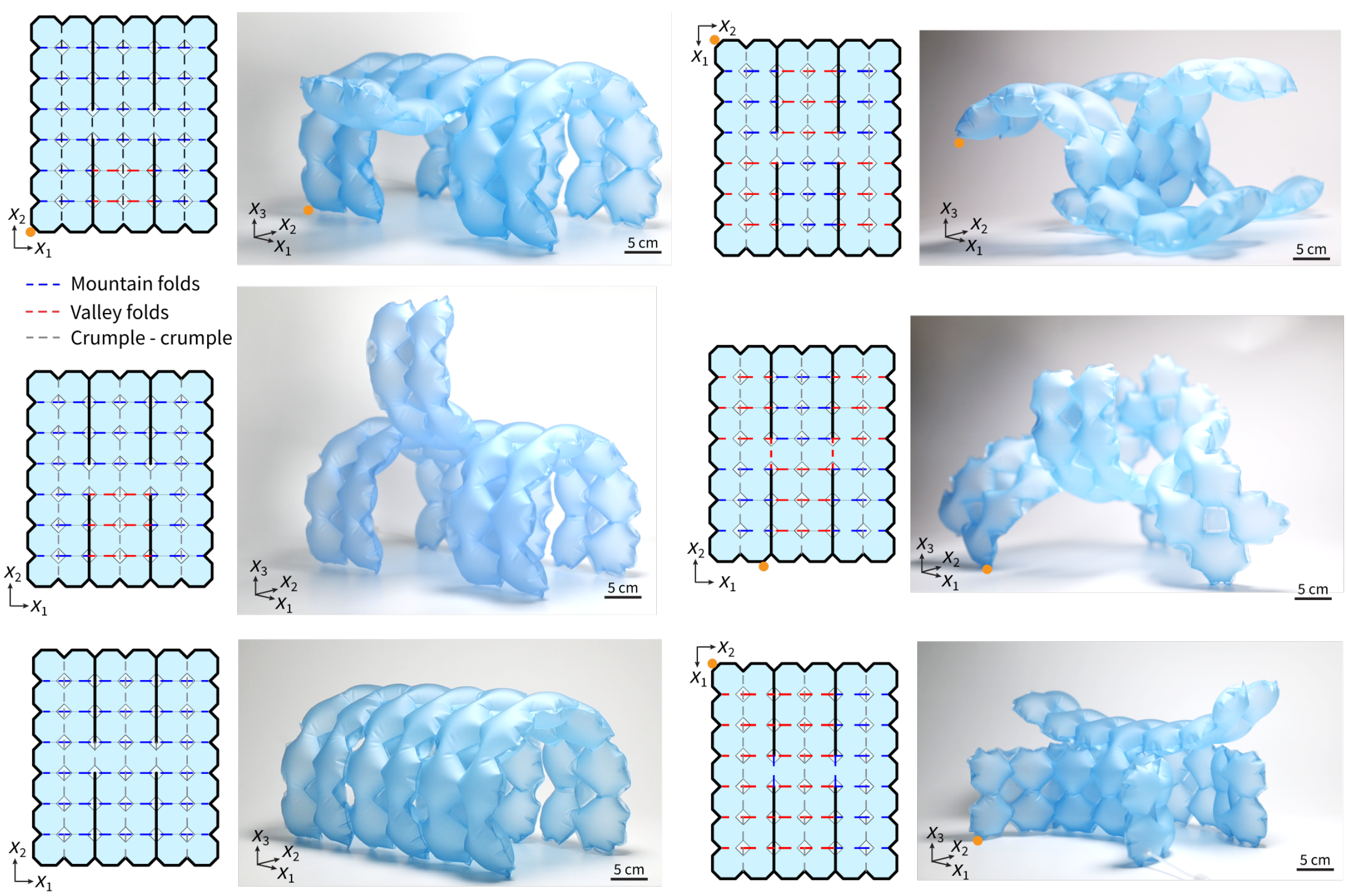

Fig. S19. 6×7 square tessellation with line cuts (Fig. 3a). Examples of stable configurations for a 6×7 square tessellation of building blocks with $a = b = 60$ mm, $s = 30$ mm, and $\theta = 90°$, incorporating four line cuts (indicated by thick black lines in the schematic). These configurations are achieved by selectively switching specific crumple lines from mountain folds to valley folds.

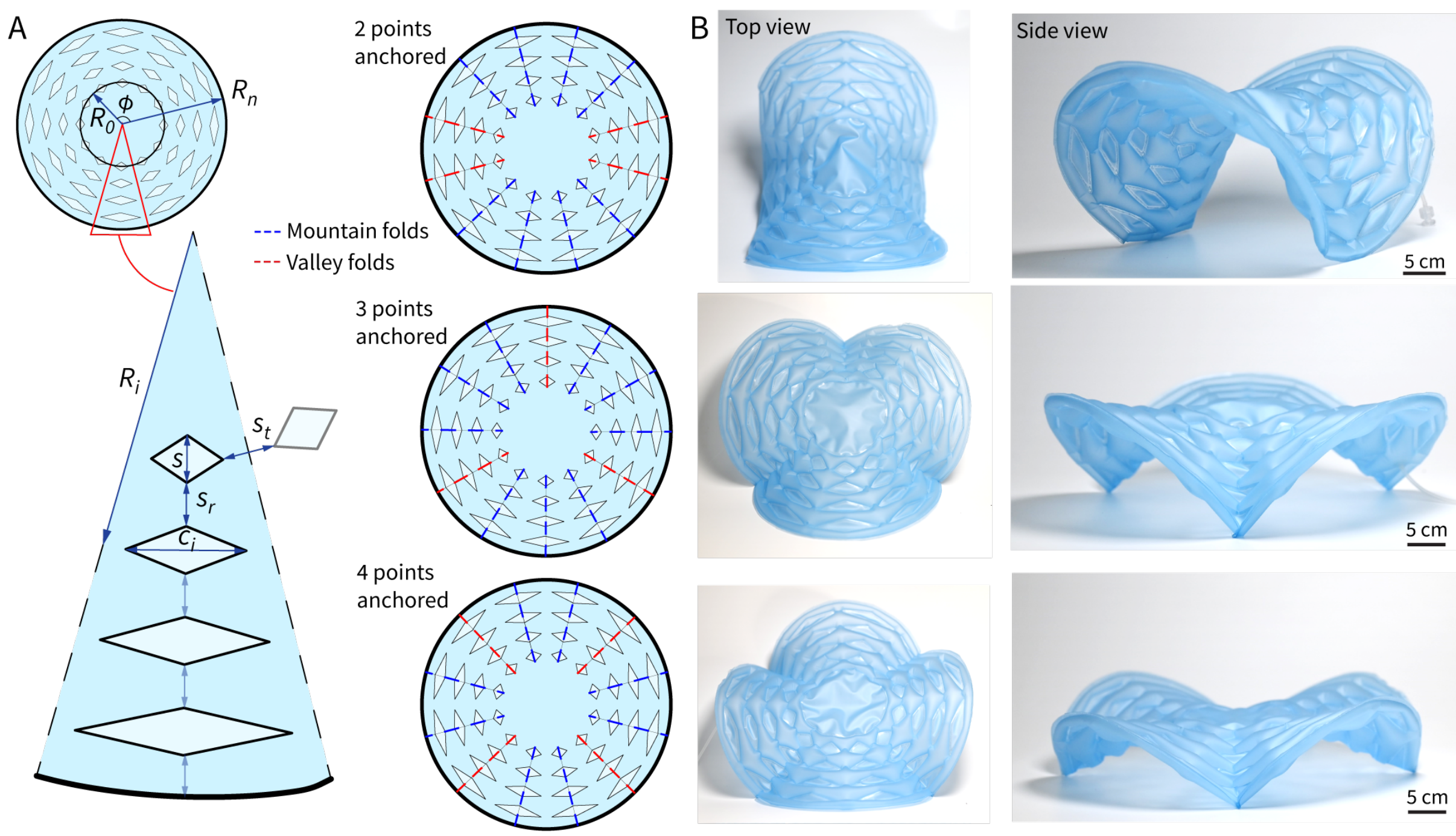

Fig. S20. Circular array of building blocks (Fig. 3b). (A) In the design considered in Fig. 3b the diamond inclusions maintain a constant vertex-to-vertex spacing of 30 mm along both radial and tangential directions ($s = s_t = s_r = 30$ mm). Furthermore, we choose the diagonal of the diamond inclusions to be aligned along the radial direction, identical to the vertex-to-vertex spacing. If n denotes the number of diamond inclusions in the radial direction, the outer radius of the disk, R_n, is given by

$$R_n = R_0 + 2n \cdot s, \tag{S16}$$

where R_0 is the radial distance for the innermost inclusions closest to the center. If m denotes the number of inclusions along the tangential direction, and c_i is the length of the tangential diagonal of the i-th inclusion located at a radial distance R_i from the center, then

$$R_i 2\pi \approx m\,[c_i + s]\,, \tag{S17}$$

where

$$R_i = R_0 + 2i \cdot s. \tag{S18}$$

The inflatable shown in Fig. 3b is designed using Eqs. (S16)-(S18) with design parameters $s = s_t = s_r = 30$ mm, $R_n = 178$ mm, $m = 12$, and $n = 4$. The tangential diagonals c_i are obtained using Eqs. (S17) and (S18) as $c_i = [44, 74.2, 104.1, 134.8]$ mm. **(B)** Snapshots of the inflatable when actuated by holding two (top), three (middle), and four (bottom) points along the boundary. At the anchored points, the hinge lines form valley folds, while the remaining hinge lines develop into mountain folds.

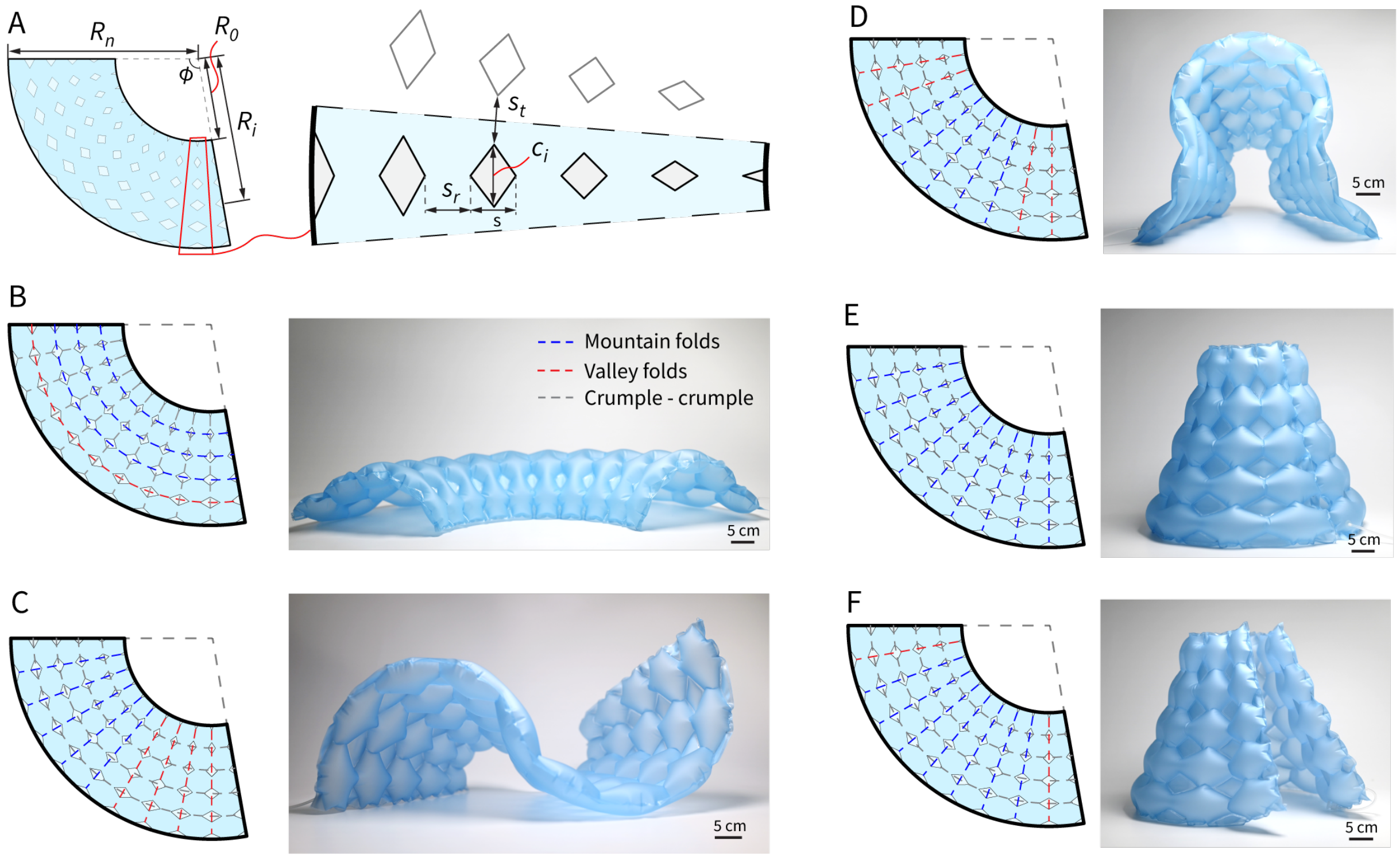

Fig. S21. Annular sector of building blocks (Fig. 3c). (A) Detailed geometry of the annular sector design shown in Fig. 3c. As in the circular design, the diamond inclusions are arranged to maintain a constant vertex-to-vertex spacing of 30 mm in both the radial and tangential directions ($s = s_t = s_r = 30$ mm). The diagonal of each diamond inclusion along the radial direction is set equal to this spacing. The geometry is determined using Eqs. , (S18), and

$$R_i \phi \approx m \left[c_i + s \right], \tag{S20}$$

where $R_0 = 250$ mm, $s = 30$ mm, $n = 5$, $m = 10$, and $\phi = 100^\circ$. The outer radius $R_n = 550$ mm is calculated from Eq. (S16), and the tangential diagonals c_i are obtained using Eqs. (S18) and (S20) as $c_i = [10.2, 19.3, 30, 41, 52.4, 70.1]$ mm. **(B)**-**(F)**) Snapshots showing five distinct stable configurations of the inflatable, accompanied by schematics indicating the corresponding locations of mountain and valley folds.

S3.3. Deployment into different stable configurations. In Figs. 2 and 3 of the main text, we present two-dimensional inflatable tessellations that can switch between multiple stable shapes. In the main text, shape switching is achieved through manual manipulation of the structure as shown in Fig. S22a; however, the deployed stable shape can alternatively be controlled by selecting appropriate boundary conditions during inflation. As an example, Fig. S22b and c show that the stable inflated shapes of the circular tessellation presented in Fig. S20 can be recovered by inflating the structure while clamping the boundary of the circular inflatable to the ground at different locations. In this demonstration, clamping is achieved by attaching the inflatable structure to linear sliders, which constrain the attachment points to planar motion along their respective axes. Upon inflation, the structure attached to two sliders forms a saddle shape with two local maxima and minima, whereas the inflatable clamped with three sliders develops three maxima and three minima. These results demonstrate that the final inflated shape can be controlled by adjusting the boundary conditions during inflation, without the need for manual manipulation after inflation. This approach is particularly well suited for large-scale structures that are difficult to handle but do not require frequent reconfiguration.

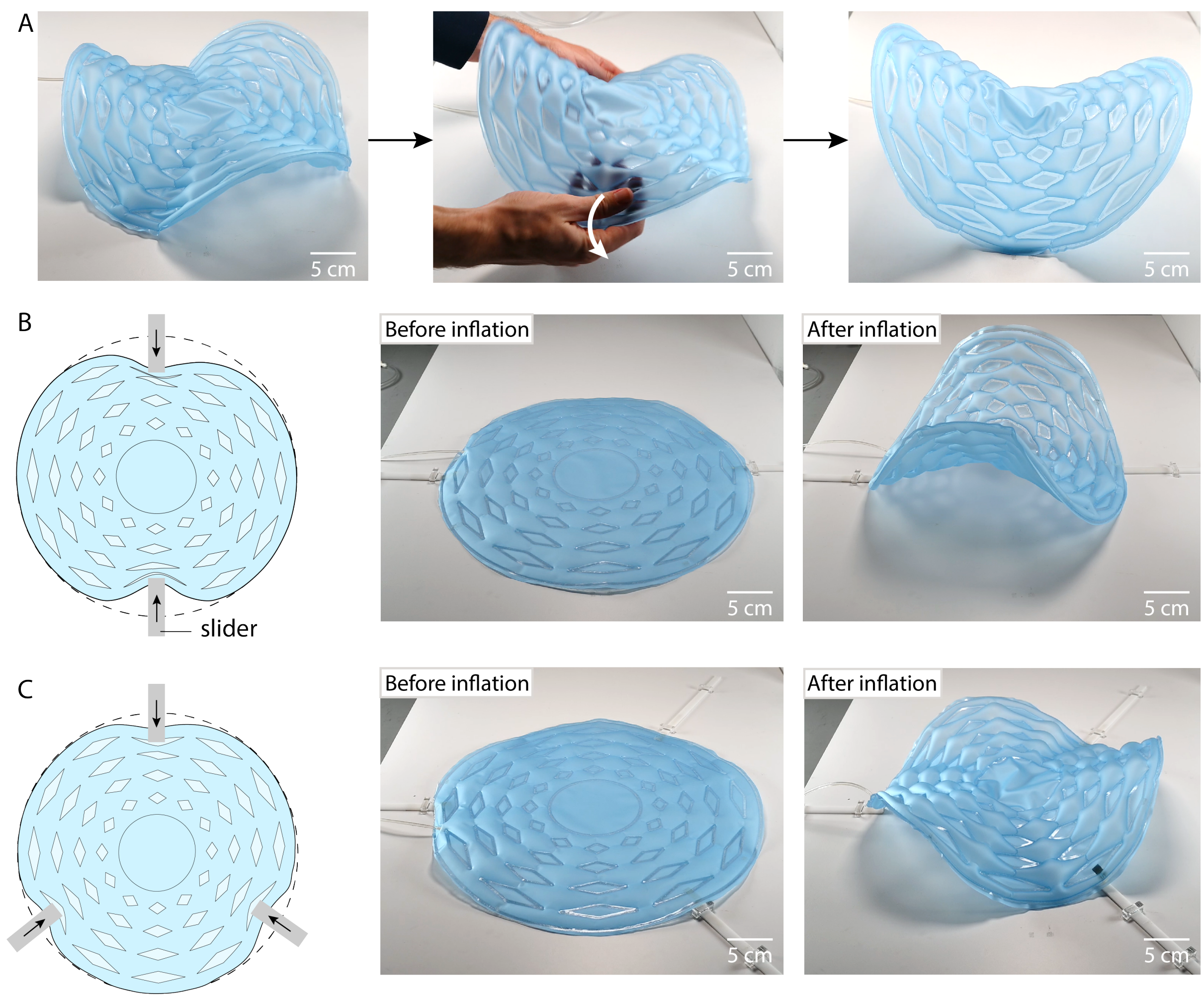

Fig. S22. Controlling the deployed stable shape via boundary conditions. (A) Snapshots showing the reconfiguration of the circular tessellation presented in Fig. S20 from three to two undulations by hand. (B)-(C) Schematics and snaphots showing the initial configuration and the resulting inflated shape of the circular tessellation with (B) two evenly spaced anchor points and (C) three evenly spaced anchor points.

S3.4. Geometric frustration in curved tessellations. The inflatables shown in Figs. 2 and 3a of the main text are patterned such that the hinge lines are aligned along straight directions, allowing bistable fold lines to form along these paths. In contrast, for certain tessellations such as the disk and annular sections presented in Figs. 3b and 3c, the hinges are not collinear but are instead oriented at angles relative to one another. This misalignment introduces geometric frustration that can inhibit the formation of continuous crease lines along the tangential direction.

To illustrate the origin of this frustration, we approximate each segment between neighboring crumpled hinges as a rigid panel connected by hinges at the crumples, as shown in Figs. S23a and S23c. The folding angle of each hinge along the tangential direction is estimated by linearly interpolating the bending angle β of the inflatable building block as a function of the notch angle θ, using the data reported in Fig. S8c. Applying these bending angles to the hinge network reveals that the

non-parallel orientation of adjacent hinges in the tangential direction leads to separation between neighboring panels. This geometric incompatibility can be accommodated by the compliance of the inflatable, since the panels in our system are not rigid but highly deformable. However, such accommodation introduces an additional energetic cost, which effectively suppresses certain otherwise stable configurations.

Figs. S23a and S23b show the rigid-panel approximation and the resulting estimated geometric frustration for the circular disk and annular section presented in Figs. S20 and S21, respectively. This analysis indicates that, if the structure were composed of rigid panels, the annular section would be unable to form an arched tunnel shape and could only adopt a conical configuration. In contrast, observations of the physical sample in Fig. S21 show that the compliant nature of the inflatable allows the structure to remain stable in the arched configuration, as the panels are not rigid but highly deformable. By comparison, a pattern with more widely spaced radial lines, such as the inflatable disk shown in Fig. S23c, exhibits substantially greater incompatibility (see Fig. S23d) and is unable to fold into an arched configuration.

The contrasting physical responses of the two structures shown in Fig. S23 highlight a limitation of the rigid-panel design approach discussed in Section S3.2. By neglecting structural compliance, this approach cannot fully evaluate the stability of different configurations. As a result, more detailed modeling, such as the Finite Element approach presented in Section S3.6, is required to accurately capture the mechanical response and assess configuration stability. In future work, we aim to accelerate these models to facilitate their direct integration into the design process for inflatable structures.

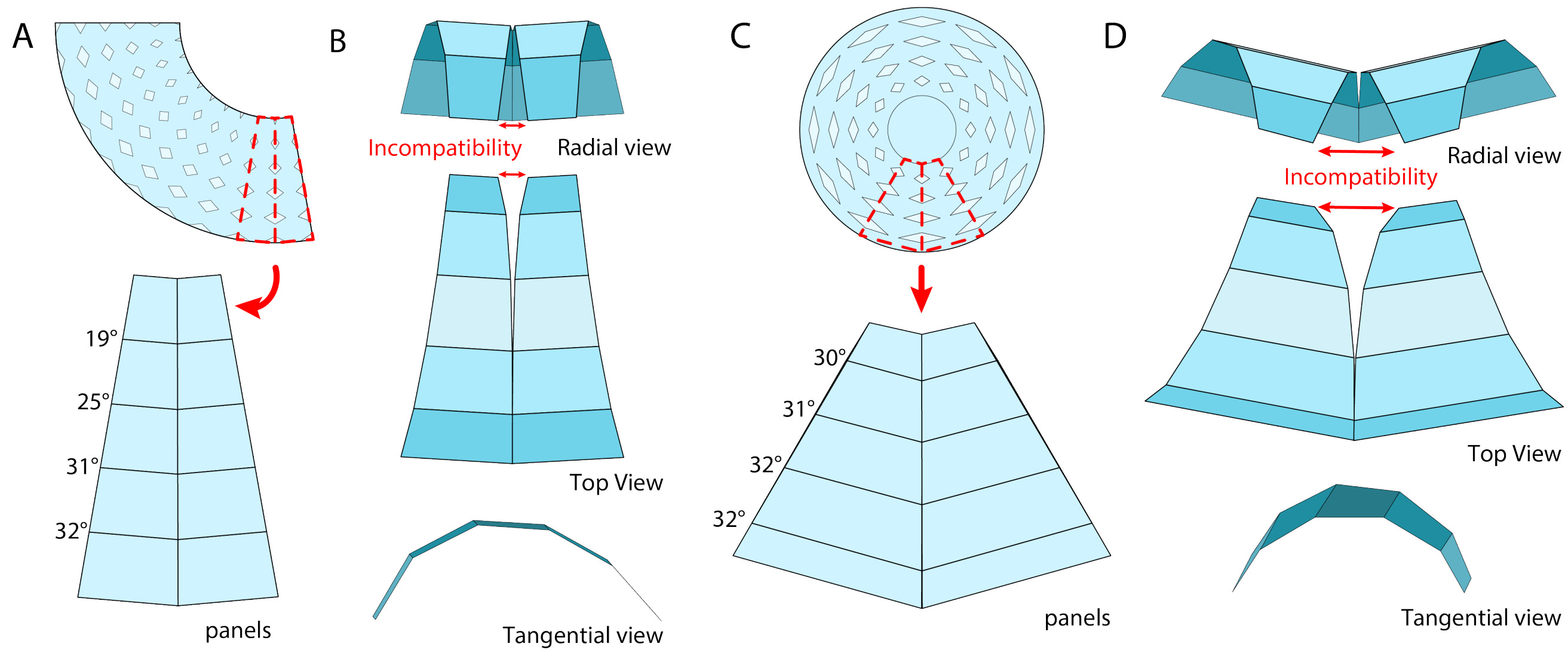

Fig. S23. Geometric frustration in circular patterns. (A) For the annular segment considered in Fig. 3c of the main text, each segment between neighboring crumpled hinges is approximated as a rigid panel connected by hinges at the crumples. (B) Estimated shape of the annular segment upon bending of the hinges along the tangential direction. (C) For the circular pattern considered in Fig. 3b of the main text, each segment between neighboring crumpled hinges is approximated as a rigid panel connected by hinges at the crumples. (D) Estimated shape of the circular pattern upon bending of the hinges along the tangential direction.

S3.5. Impact mitigation. Here, we describe the experimental setup for the drop tests, as well as the reduced-order model used to predict the drop height required to trigger snapping for a given object mass.

Experimental Setup To quantify the energy absorption and impact mitigation capabilities of our inflated 2D tessellations, we conduct drop-weight impact tests. In these tests, a 3D-printed sphere (Formlabs Form 3B) with a mass of 122 grams is released from a height H above the apex of the inflated sample. The multistable inflatable is first inflated to a pressure of 6 kPa and then clamped along two opposite edges (Fig. S24). To ensure a controlled and repeatable drop, the sphere slides along a low-friction guide rod (10 mm in diameter) positioned above the center of the multistable inflatable sample (Fig. S24). Tracking dots are placed on the sphere, and its motion is recorded using a high-speed camera (Phantom TMX 7510) at a frame rate of 2000 fps. Image processing is then performed using a custom MATLAB code to extract the sphere's position over time, from which velocity and acceleration are numerically computed.

Analytical model To predict the drop height required for the multistable inflatable to snap into its inverted stable configuration, we develop a simple analytical model. In this model, the inflatable is represented as a nonlinear spring in parallel with a linear dashpot (Fig. S25). We focus on the time interval between the moment the dropped object makes contact with the inflatable and the moment it detaches. During this period, the object and inflatable move together, and the system can be modeled as a mass–spring–dashpot system subjected to an initial velocity $v_0 = \frac{du}{dt}(0) = \sqrt{2gH}$, where H is the drop height. The equation of motion governing the system is:

$$m\frac{d^2u}{dt^2} + c\frac{du}{dt} + F_s(u) = mg \qquad [S21]$$

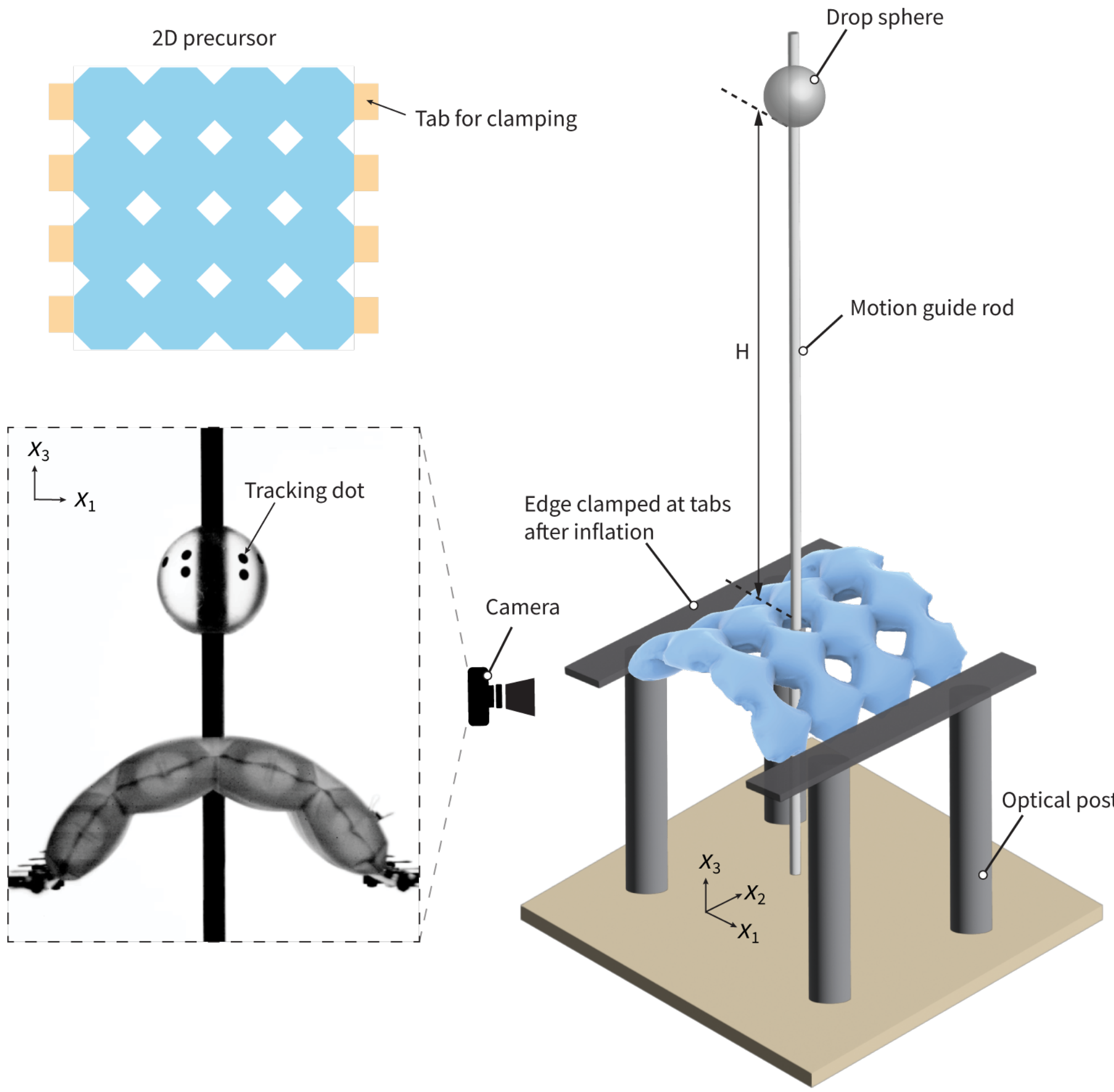

Fig. S24. Experimental setup. Schematic of the setup used to quantify the energy absorption and impact mitigation capabilities of the inflated 2D tessellations.

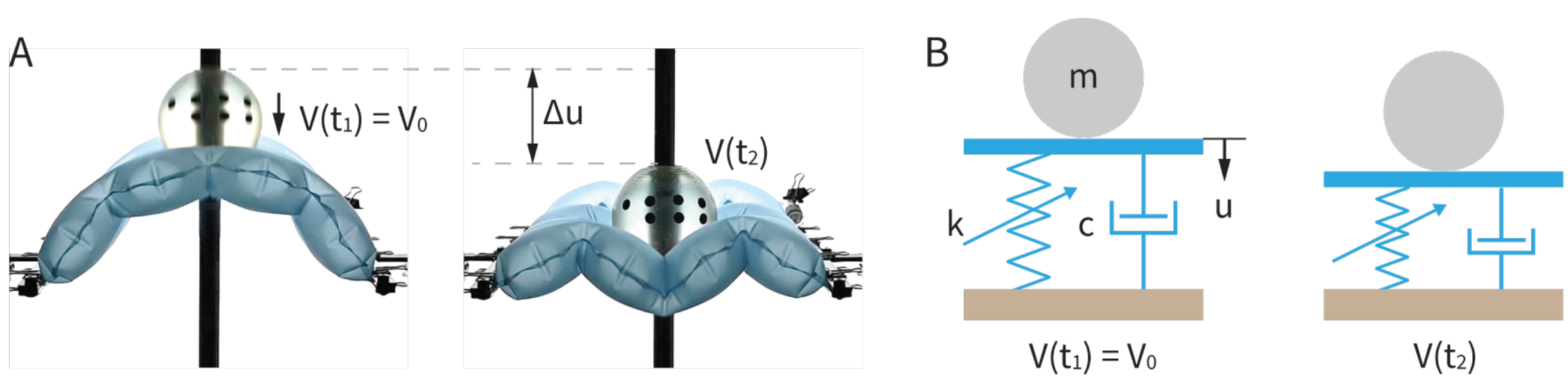

Fig. S25. Analytical model. A, Experimental images showing the initial impact of the sphere with the inflatable and the moment just before snapping occurs. **A**, Schematic of the simplified analytical model.

where $F_s(u)$ is the nonlinear force–displacement response of the spring, and c is the damping coefficient.

To characterize $F_s(u)$, we perform displacement-controlled loading tests on inflatables pressurized at $p = 6$ kPa, using the same sphere from the impact experiments as the indenter head mounted on an Instron 5969 system (Fig.S26A). To assess the influence of displacement rate, we conduct tests at three rates: 0.1 mm/s, 0.5 mm/s, and 5 mm/s. The corresponding force–displacement curves are shown in Fig.S26B. As expected from viscoelastic behavior, the measured force increases with displacement rate. For modeling purposes, we use the intermediate rate of 0.5 mm/s to define the nonlinear spring response and fit the experimental force–displacement data with a 7th-order polynomial using MATLAB's Curve Fitting Toolbox (MathWorks, Inc.):

$$F_s(u) = k_1 u^7 + k_2 u^6 + ...k_7 u + k_8 \quad \text{[S22]}$$

where, $F_s(u)$ is the nonlinear force measured in N, u is the displacement measured in mm, and the spring coefficients are $k_i = [-5.56 \times 10^{-10}, 1.064 \times 10^{-7}, -8 \times 10^{-6}, 2.99 \times 10^{-4}, -5.7 \times 10^{-3}, 0.0488, 0.0779, 0.1218]$ N/mm. As shown in Fig. S26C,

the fitted polynomial accurately captures the experimentally measured force–displacement curve.

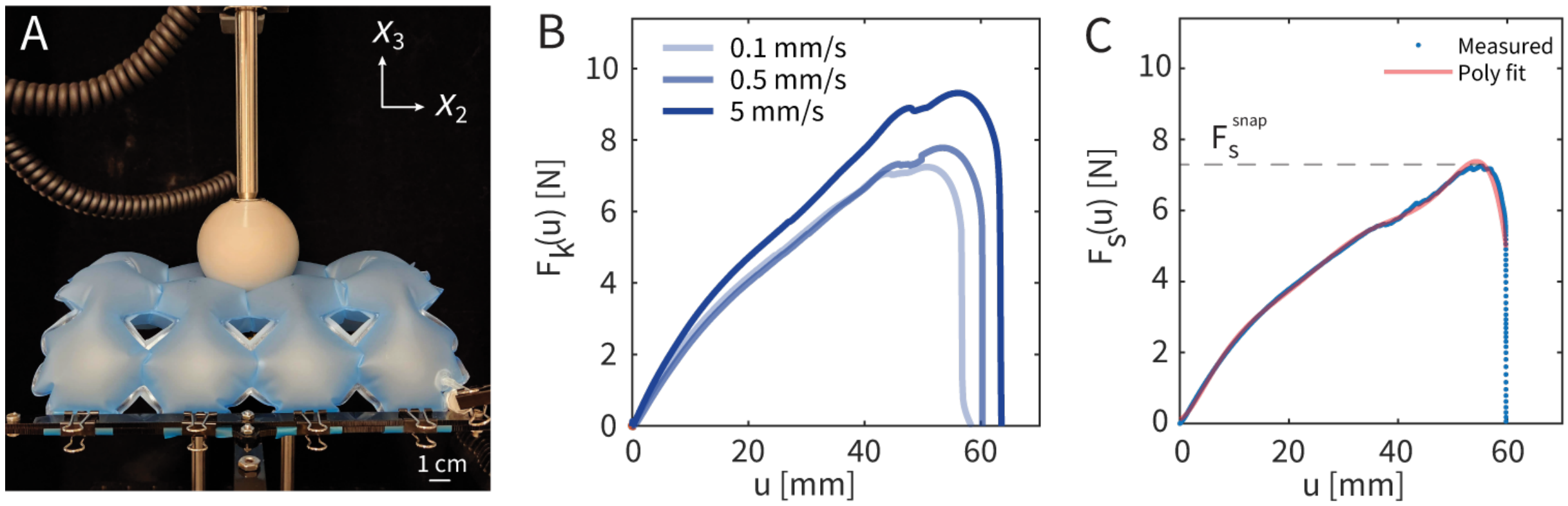

Fig. S26. Characterization of nonlinear force-displacement response. a, Images of the experimental setup. **b**, Measured force-displacement curves at displacement rates of 0.5 mm/s, 1.0 mm/s, and 5.0 mm/s. **c**, Comparison between experimentally measured force-displacement curves and fit using a 7th-order polynomial.

Next, to estimate the damping coefficient c, we perform free vibration tests on the inflatable pressurized at $p = 6$ kPa. As shown in Fig. S27, a weight is attached to the top of the inflatable and vertically displaced by approximately 10 mm before being suddenly released. The resulting oscillations are tracked by monitoring the vertical position of the weight over time using a digital camera (Nikon Z6). In Fig. S27, we report the experimentally measured displacement–time curve for a weight of $m = 0.2$ kg, and observe that the system exhibits underdamped behavior characteristic of a mass–spring–dashpot system. In this regime, the envelope of the oscillation follows an exponential decay of the form (4):

$$x(t)\Big|_{\text{envelope}} = A_0 \exp\left(-\frac{c}{2m}t\right) \quad \text{[S23]}$$

We estimate the damping coefficient c by fitting this expression to the experimental data (see red curves in Fig. S27). The tests are repeated using three different weights: $m = 0.2$ kg, 0.25 kg, and 0.3 kg. The best-fit values for the damping coefficient are $c = 1.97$ kg/s, 2.04 kg/s, and 2.46 kg/s, respectively. We take the average of these values and use $c = 2.16$ kg/s as the effective structural damping coefficient in our model.

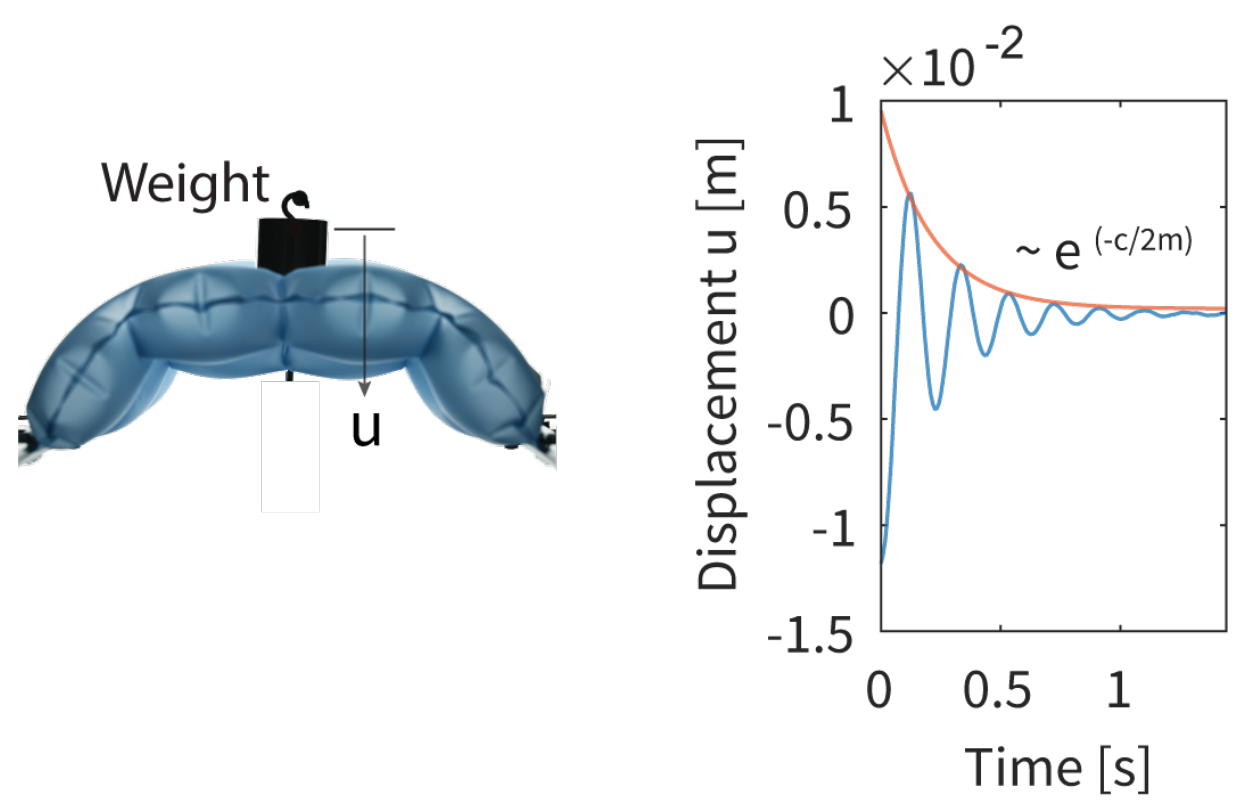

Fig. S27. Characterization of viscous damping. Snapshot of the inflatable with an attached weight and its corresponding recorded displacement of the weight after release of the inflatable, following a downward displacement of $u \approx 10$, mm.

Having determined both $F_s(u)$ and c, we numerically integrate Eq. (S21) using MATLAB's 'ode45' solver (MathWorks, Inc.), with initial conditions $u(0) = 0$ and $v_0 = \frac{du}{dt}(0) = \sqrt{2gH}$. We progressively increase the drop height H, solve Eq. (S21) for each value, and monitor whether the force in the spring reaches the snapping threshold $F_s^{\text{snap}} = 7.25$ N. If the force reaches this threshold, we assume that the inflatable has snapped into its inverted configuration; otherwise, we consider that snapping has not occurred. The results of this analysis are reported in Fig. 4g of the main text.

S3.6. Finite Element simulations. To demonstrate that the FE framework described in Section S2.5 can be used as a design and analysis tool for multistable tessellated structures, we simulated the tessellated geometry shown in Fig. 2 of the main text. The modeling approach follows the methodology described in Section S2.5; in particular, the same element types, material model, and numerical setup are employed in these simulations.

We construct a FE model of the tessellated structure consisting of a 4×4 square array of building blocks, as investigated in Fig. 2 of the main text. To suppress rigid-body motion while preserving the global deformability of the tessellated structure, displacement boundary conditions are applied only at three corner nodes as shown in Fig. S28A. All remaining nodes are unconstrained.

The inflation of the model is simulated using an implicit dynamic analysis, consistent with Section S2.5. The loading protocol consists of three steps:

- *Inflation:* The internal cavity of the tessellated structure is inflated using a fluid cavity with volume control. The prescribed cavity volume is increased incrementally until an internal pressure of approximately 6 kPa is reached. Fig. S28B shows the configuration of the inflatable after this step.
- *Mechanical manipulation:* Starting from the inflated equilibrium configuration, displacement boundary conditions are applied at selected nodes to reconfigure the structure into an alternative stable state. These displacements are chosen to promote the global mode switch observed experimentally and discussed in the main text. The displacements are then held constant for a short duration to allow dynamic convergence (*hold step*) before being released.
- *Relaxation:* Following the removal of the applied displacements, the structure is allowed to relax freely and reach a stable configuration. Depending on the spatial location of the applied actuation, different stable configurations of the tessellated structure can be accessed.

Starting from the inflated configuration, different stable configurations can be reached depending on the location at which the mechanical actuation is applied during the second step. This enables controlled transitions between multiple equilibrium states of the tessellated structure, resulting in either an W-shaped global configuration (Fig. S28C) or a U-shaped global configuration (Fig. S28D), in agreement with the experimentally observed states shown in Fig. 2 of the main text.

The multistable behavior of the tessellated structure is further illustrated by the evolution of the strain and kinetic energies. The strain energy exhibits distinct local minima following the relaxation step (Fig. S28E). Correspondingly, the kinetic energy decays to negligible values (Fig. S28F), indicating that the system has reached stable equilibrium configurations. The simulations recover the same stable states observed experimentally, confirming that the proposed FE framework accurately captures both the inflation-induced deformation and the mechanically triggered state transitions of tessellated multistable structures.

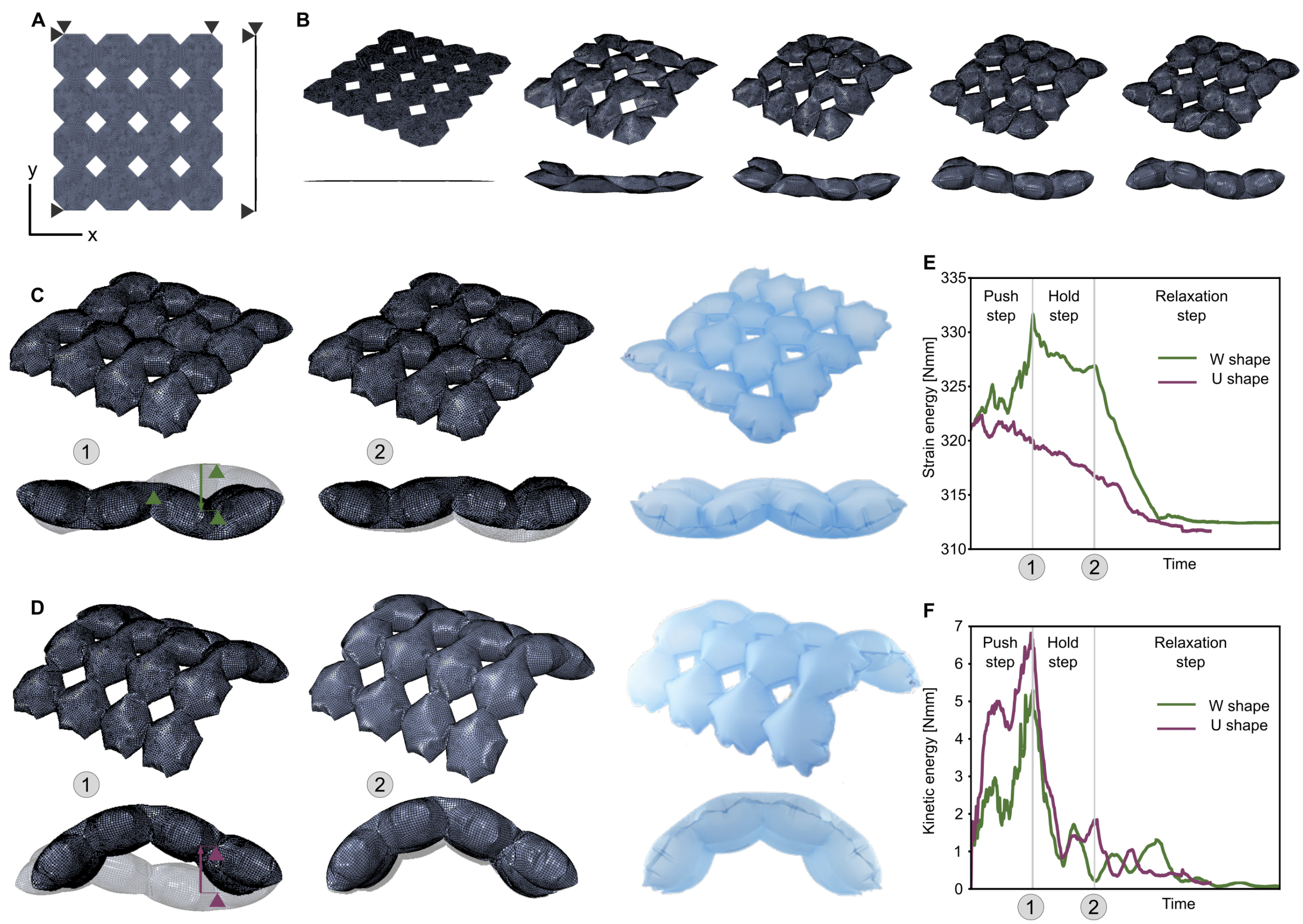

Fig. S28. Finite-element simulation of a tessellated multistable structure. (A) Top view of the 4×4 tessellated FE model with applied boundary conditions. (B) Deformation sequence during the volume-controlled inflation stage until $6\,\mathrm{kPa}$. (C) W-shaped stable configuration with locations and directions of the applied mechanical actuation during the push step. (D) U-shaped stable configuration with locations and directions of the applied mechanical actuation during the push step. (E) Strain energy during the push, hold, and relaxation steps for both W-shaped and U-shaped configurations. The local minima correspond to stable equilibrium states. (F) Corresponding kinetic energy, demonstrating dynamic convergence during the hold and relaxation steps.

S4. Captions of Supporting Videos

Movie S1. Bistability via crumpling

Crumples emerge between the notches of our rectangular inflatable building block, giving rise to bistability.

Movie S2. Multistable inflatable tessellations

Our bistable inflatable building blocks can be assembled into multistable inflatables capable of transitioning between a variety of stable shapes. We show multistability in three configurations: a one-dimensional array, a square two-dimensional array, and a triangular array of building blocks. In all the tessellations considered, each building block is defined by the parameters $a = b = 60$ mm, $s = 30$ mm, and $\theta = 90^\circ$.

Movie S3. Reconfigurable multistable inflatables at the centimeter scale

Complex stable shapes are demonstrated in three configurations: a 6×7 square array of building blocks incorporating four cuts; a disk tessellated with the building block; and a 10×5 array of diamond-shaped, non-inflating regions arranged within an annular sector.

Movie S4. Reconfigurable multistable inflatables at the meter scale

The capability to construct lightweight inflatable structures that can be reconfigured into tunnels and canopies with adjustable openings opens up exciting possibilities for the design of large-scale, reconfigurable systems. This supplementary video includes footage of a bistable, meter-scale building block, as well as a meter-scale inflatable identical in design to that shown in Fig. 3c of the main text, but scaled up by a factor of ten.

Movie S5. Multistable inflatable for impact mitigation

In addition to enabling shape-shifting capabilities, multistable inflatables offer promising potential for the development of advanced impact-mitigation systems. This supplementary video includes footage of drop tests conducted on both the multistable and monostable inflatables.

References

1. S. L. Veldman, O. K. Bergsma, and A. Beukers. Bending of anisotropic inflated cylindrical beams. *Thin-Walled Structures*, 43:461–475, 2005. .
2. L. G. Brazier. On the flexure of thin cylindrical shells and other thin sections. *Proceedings of the Royal Society of London. Series A*, 116(773):104–114, 1927. .
3. Yue Yang, Lei Ren, Chuang Chen, Bin Hu, Zhuoyi Zhang, Xinyan Li, Yanchen Shen, Kuangqi Zhu, Junzhe Ji, Yuyang Zhang, Yongbo Ni, Jiayi Wu, Qi Wang, Jiang Wu, Lingyun Sun, Ye Tao, and Guanyun Wang. Snapinflatables: Designing inflatables with snap-through instability for responsive interaction. In *Proceedings of the 2024 CHI Conference on Human Factors in Computing Systems*, CHI '24, New York, NY, USA, 2024. Association for Computing Machinery. ISBN 9798400703300. . URL https://doi.org/10.1145/3613904.3642933.
4. Singiresu S Rao. *Mechanical Vibrations*. Prentice Hall, 2011.
